# Supplementary material for: Individual-level surrogacy of MRI lesions for disease severity in RRMS: Methods to quantify predictive power and their application to longitudinal data from recent trials
Source: PLoS One. 2025 Dec 26;20(12):e0337893. doi: 10.1371/journal.pone.0337893 (PMC12742783; doi:10.1371/journal.pone.0337893)
Supplement: S1 Text — (DOCX) [file pone.0337893.s001.docx]

**S1 text: Diverse supportive information**

**Content**

[1. Prentice criteria and Proportion of Treatment Effect Explained (PTE) 2](#_Toc212542414)

[2. Information-theoretic approach 9](#_Toc212542415)

[3. Example: Quantifying information shared between two binary variables using mutual information 13](#_Toc212542416)

[4. PTE, Likelihood ratio test, and $\mathbf{Rhindiv^{2}}$: an illustration of group-based versus individual-level, variance-reduction-based surrogacy metrics. 21](#_Toc212542417)

[5. Relationship between positive/negative predictive values considering SEP/CEP prevalence. 25](#_Toc212542418)

[References 31](#_Toc212542419)

**Figures**

[**Figure A:** Relationship between surrogate endpoint and clinical endpoint (CEP): low variability in CEP. LRF, Likelihood Reduction Factor; PTE, Proportion of Treatment Effect explained; R², coefficient of determination of a linear regression model between CEP and the surrogate adjusted for treatment. 4](#_Toc212542814)

[**Figure B:** Relationship between surrogate endpoint and clinical endpoint (CEP): high variability in CEP. LRF, Likelihood Reduction Factor; PTE, Proportion of Treatment Effect explained; R², coefficient of determination of a linear regression model between CEP and the surrogate adjusted for treatment. 5](#_Toc212542815)

[**Figure C*:*** Effect of SEP/CEP variance on several prediction performance metrics (when SEP/CEP inducing association $\alpha=1.5$) 21](#_Toc212542816)

[**Figure D:** Positive Predictive Value and Negative Predictive Value considering different prevalences 27](#_Toc212542817)

**Tables**

[**Table A:** Definition of the regression models shown in **Figure A** and **Figure B** 6](#_Toc212542840)

[***Table B:*** *Summary of shown scenarios* 26](#_Toc212542841)

## **1.** **Prentice criteria and Proportion of Treatment Effect Explained (PTE)**

1. **Definition**

In 1989, Prentice proposed four criteria that a variable must meet to qualify as a surrogate endpoint (SEP) ^1^. These criteria can be evaluated using statistical models:

1. The SEP depends on the treatment Z.
2. The CEP depends on the treatment Z.
3. The SEP has information about CEP.
4. The SEP contains all the information of the treatment effect.

**Equation A: Models for Prentice criteria:**

1. $f\left( \mathrm{SEP} | Z \right)\neq f\left( \mathrm{SEP} \right):\mathrm{SEP}_{j}=\mu_{s}+\alpha Z_{j}+\epsilon_{\mathrm{Sj}}$
2. $f\left( \mathrm{CEP} | Z \right)\neq f\left( \mathrm{CEP} \right):\mathrm{CEP}_{j}=\mu_{T}+\beta Z_{j}+\epsilon_{\mathrm{Tj}}$
3. $f\left( \mathrm{CEP} | \mathrm{SEP} \right)\neq f\left( \mathrm{CEP} \right) : \mathrm{CEP}_{j}=\mu+\gamma S_{j}+\epsilon_{j}$
4. $f\left( \mathrm{CEP} | SEP,Z \right) = f\left( \mathrm{CEP} | \mathrm{SEP} \right): \mathrm{CEP}_{j} =\mu_{T} + \beta_{S} Z_{j} + \gamma_{Z} \mathrm{SEP}_{j} +\epsilon_{T}$*,*

where:

- $j$ = 1, 2, …, n subjects
- $\mathrm{SEP}$: Surrogate endpoint
- $\mathrm{CEP}$: Clinical endpoint
- $Z$: Treatment indicator
- $\mu,\mu_{s},\mu_{T}$: Model specific intercepts
- $\alpha:$ Treatment effect on $\mathrm{SEP}$.
  To fulfill criterion 1, this estimate must be **significant**
- $\beta:$ Treatment effect on $\mathrm{CEP}$
  To fulfill criterion 2, this estimate must be **significant**
- $\gamma:$ Effect of SEP on $\mathrm{CEP}$
  To fulfill criterion 3, this estimate must be **significant**
- $\beta_{S}:$ Treatment Effect when $\mathrm{SEP}$ is an independent variable in the model (Treatment effect captured by $\mathrm{SEP}$).
  To fulfill criterion 4, this estimate must be **non-significant.**

The Prentice criteria are the basis to define the PTE (Proportion of the treatment effect explained). For more details see the following section. Often Prentice Criteria and PTE are considered as expressing the same message regarding the surrogacy of the SEP for the CEP.

**Proportion of Treatment effect Explained (PTE):**

In 1992, Freedman introduced the Proportion for Treatment effect Explained (PTE) ^2^

**Equation B: Estimation of the proportion treatment effect explained (PTE)**

$\mathrm{PTE}\left( CEP,SEP,Z \right)=\frac{\beta-\beta_{S}}{\beta}=1-\frac{\beta_{S}}{\beta}$,

where:

- $\beta:$ Treatment effect on $\mathrm{CEP}$ (related to Prentice criterion 2)
- $\beta_{S}:$ Treatment effect, when $\mathrm{SEP}$ is an independent variable in the model (Treatment effect captured by SEP. related to Prentice criterion 4)

$\beta$ and $\beta_{s}$ are the estimates from the regression equations presented in Prentice criteria 2 and 4 as described in ***Equation A***.

The analysis of the main article includes sensitivity analysis of the calculation of PTE values. We compare the PTE values based on our data to compare the results to existing literature. It is of interest to see if we get similar values in our setting as reported from other settings.

CEP was the number of relapses and SEP the number of new or newly enlarged T2 lesions. To this end, we used the same methodology as Sormani et al. ^3^: A negative binomial model family to fit the models proposed in the prentice criteria.

1. **Is the Proportion of Treatment effect Explained (PTE) a good measure for surrogacy?**

Look at the following setting and relationship between an SEP and a CEP:


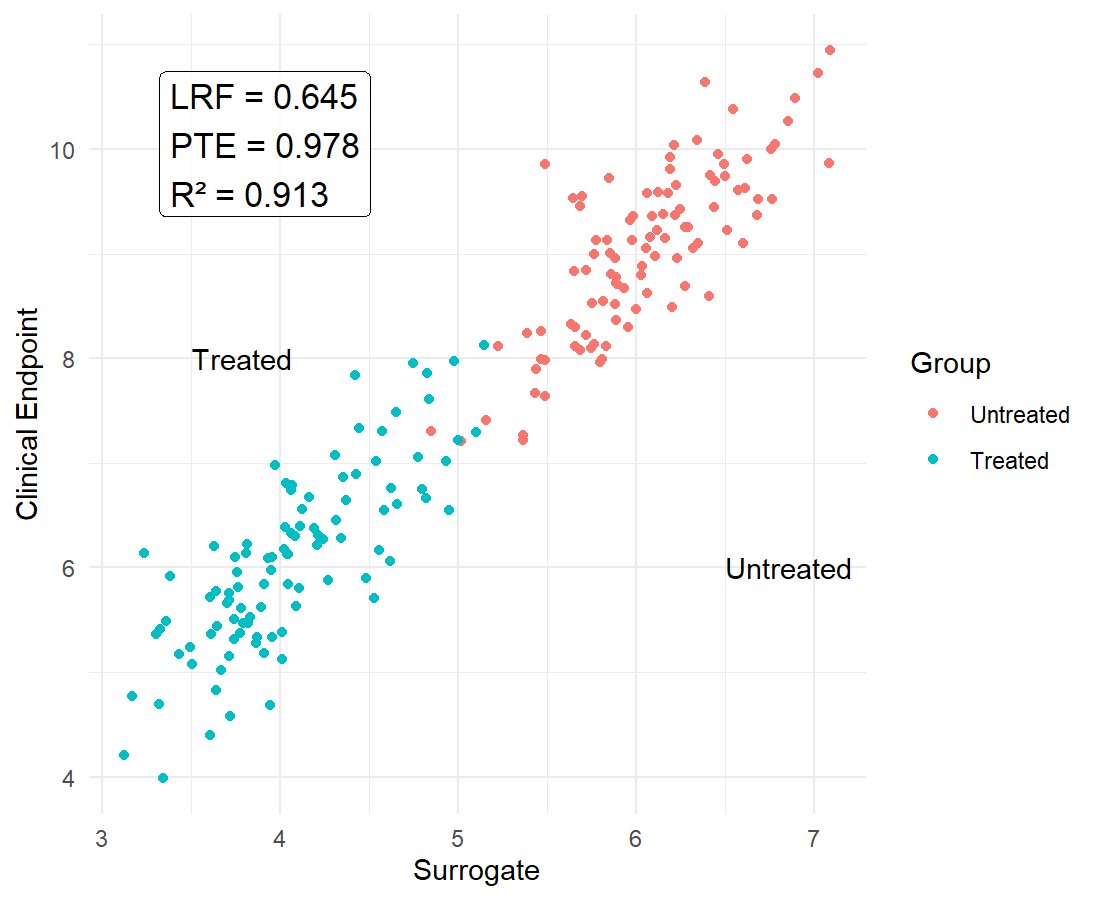


**Figure A:** Relationship between surrogate endpoint and clinical endpoint (CEP): low variability in CEP. LRF, Likelihood Reduction Factor; PTE, Proportion of Treatment Effect explained; R², coefficient of determination of a linear regression model between CEP and the surrogate adjusted for treatment.

**Figure A** shows that the SEP value depends on both treatment status and the CEP value. This satisfies criteria 1 and 2 of the Prentice criteria (see **Equation A**). In addition, there is a linear relationship between SEP and CEP, fulfilling criterion 3 via regression analysis. Criterion 4, which involves the regression of the outcome on both treatment and SEP, yields the same regression as in criterion 3 and is therefore also fulfilled. Since the variability between SEP and CEP is small, knowledge of the surrogate allows for reasonably accurate predictions of the clinical endpoint in both treated and untreated patients. For example, when the SEP value is 6, the corresponding CEP values in treated individuals typically fall within the range of 7 to 10. In this scenario, the proportion of treatment effect explained (PTE) can be calculated and equals 1.

Now we consider the same regression models as before, but with increased variability in the CEP. This clearly reduces the quality of the surrogacy between CEP and SEP. The predictions become less accurate, and our certainty about the CEP given the SEP decreases. However, despite this decline in predictive precision, the PTE remains theoretically unchanged and equals 1 in both scenarios.


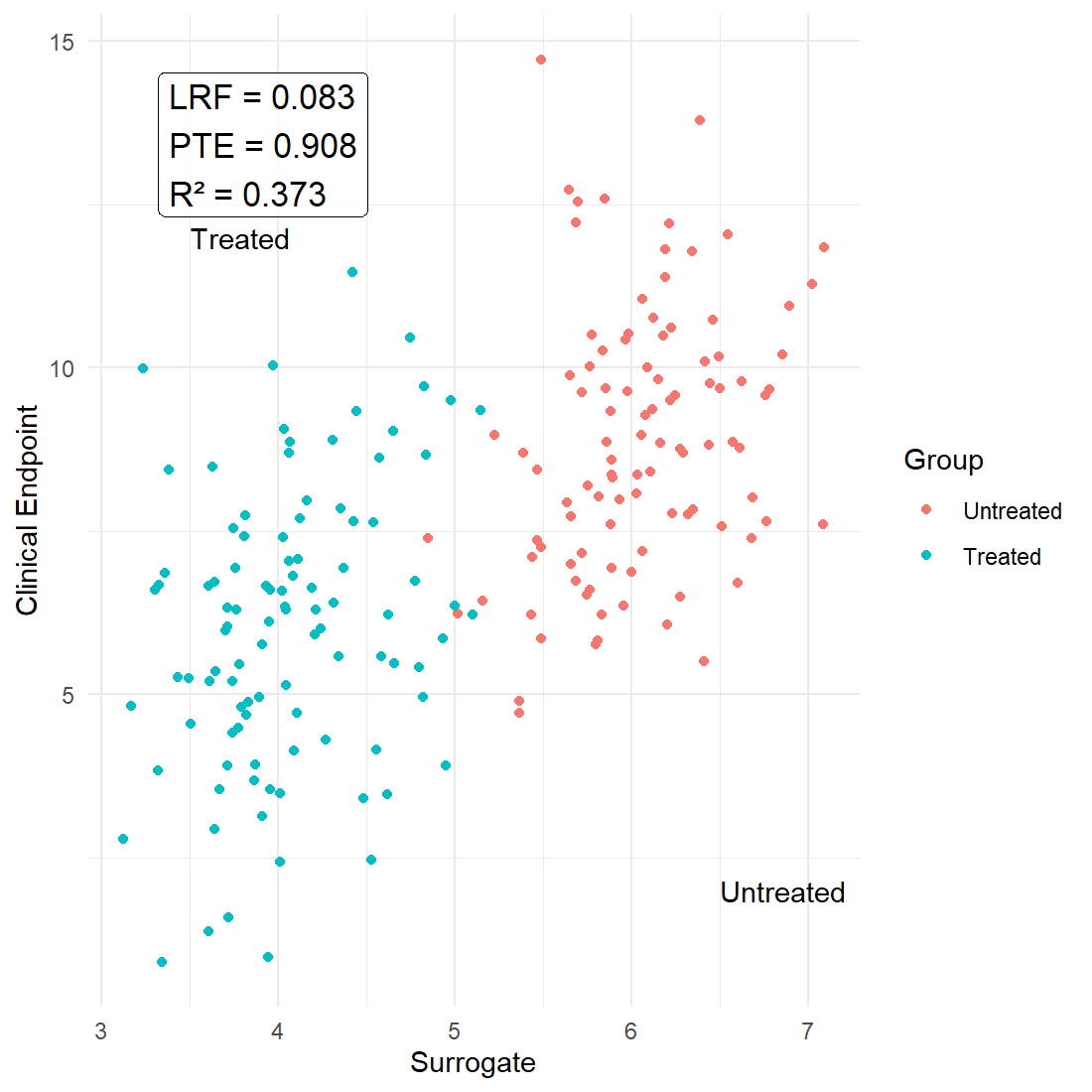


**Figure B:** Relationship between surrogate endpoint and clinical endpoint (CEP): high variability in CEP. LRF, Likelihood Reduction Factor; PTE, Proportion of Treatment Effect explained; R², coefficient of determination of a linear regression model between CEP and the surrogate adjusted for treatment.

Formally, we have the following regression defined

| **Criteria** | **Setting 1 (STD:** $\boldsymbol{\varepsilon}_{\boldsymbol{1}}\boldsymbol{=0.5,}\boldsymbol{\varepsilon}_{\boldsymbol{2}}\boldsymbol{=0.5}$**)** | **Setting 2 (**$\mathbf{STD}\boldsymbol{:}\boldsymbol{\varepsilon}_{\boldsymbol{1}}\boldsymbol{=0.5,}\boldsymbol{\varepsilon}_{\boldsymbol{2}}\boldsymbol{=1.5}$**)** |
| --- | --- | --- |
| $\boldsymbol{SEP}\boldsymbol{\sim}\boldsymbol{Z}$ | $6-2\cdot Z+\varepsilon_{1}$ | $6-2\cdot Z+\varepsilon_{1}$ |
| $\boldsymbol{CEP}\boldsymbol{\sim}\boldsymbol{Z}$ | $9-3\cdot Z+\varepsilon_{2}$ | $9-3\cdot Z+\varepsilon_{2}$ |
| $\boldsymbol{CEP}\boldsymbol{\sim}\boldsymbol{SEP}$ | $1.5\cdot\text{SEP}+\varepsilon_{3}$ | $1.5\cdot\text{SEP}+\varepsilon_{4}$ |
| $\boldsymbol{CEP}\boldsymbol{\sim}\boldsymbol{SEP + Z}$ | $1.5\cdot\text{SEP}+\varepsilon_{3}$ | $1.5\cdot\text{SEP}+\varepsilon_{3}$ |
| **PTE** | $1\left( since \beta_{S}=0 \right)$ | $1\left( since \beta_{S}=0 \right)$ |

**Table A:** Definition of the regression models shown in **Figure A** and **Figure B**

## **2. Information-theoretic approach**

A method to validate SEPs on the individual level is the information-theoretic approach (IT), which uses the Mutual Information (MI) estimated by the likelihood ratio test statistics to estimate the information, which is captured, when SEP becomes available^4^.

**Equation C: Mutual information (MI) of two random variables X and Y**

$\mathrm{MI}\left( X,Y \right)=H\left( Y \right)-H\left( Y | X \right)$, where $H$ is the Shannon entropy.

Consider the following two models:

**Equation D: Reduced model (pure treatment effect on the CEP)**

$$\mathrm{gC}\left[ E\left( \mathrm{CEP} \right) \right]=\mu_{\mathrm{Ti}}+\beta_{i}Z_{\mathrm{ij}}$$

**Equation E: Full model (treatment and SEP as covariates)**

$gC|S\left[ E\left( \mathrm{CEP}_{\mathrm{ij}} | \mathrm{SEP}_{i}j \right) \right]=\gamma_{0i}+\gamma_{1i}Z_{\mathrm{ij}}+\gamma_{2i}S_{\mathrm{ij}}$,

where $\mathrm{gC}$and $gC|S$are function to link the expectation value to the linear predictor.

In case of linear regression models with respective residuals, Alonso introduced a new association measure $R_{\mathrm{hindiv}}^{2}$:

**Equation F: Calculation of** $R_{hindiv}^{2}$ **and the likelihood reduction factor**

$R_{\mathrm{hindiv}}^{2}=1-e^{-2MI\left( \epsilon_{S},\epsilon_{C} \right)}$,

where $\epsilon_{S}$ and $\epsilon_{C}$ are correlated error terms of $\mathrm{SEP}$ and $\mathrm{CEP}$.

The $R_{\mathrm{hindiv}}^{2}$ can also be estimated using the likelihood ratio test statistics. Note, the Likelihood Reduction Factor (LRF) is based on longitudinal data, while $R_{\mathrm{hindiv}}^{2}$ is for normally distributed SEP and CEP measured only once. Hence, to estimate the LRF, both the reduced and full models defined in **Equation D** and **Equation E** must be capable of handling longitudinal data.

**Equation G: Estimation of** $R_{hindiv}^{2}$ **and the likelihood reduction factor using likelihood ratio statistics**

$$\hat{R_{\mathrm{hindiv}}^{2}}=LRF=1-\frac{1}{N}\sum_{i} e^{-\frac{G_{i}^{2}}{n_{i}}},$$

*with:*

$\frac{G^{2}}{n}=MI\left( X,Y \right)=\frac{1}{n}\sum\left( \log\left[ f\left( y_{i} | xi,\hat{\theta} \right) \right]-log\left[ f\left( y_{i} | \hat{\theta_{0}} \right) \right] \right)$,

and with:

- density function $f\left( X,Y | \theta\right)$
- realizations of $\left( x_{i},y_{i} \right),$ $i = 1,2,\ldots,n$
- parameter $\theta$ has form $\theta=\left( \theta_{0},\theta_{1} \right)$
- $\hat{\theta_{0}}$ = maximum likelihood estimate (under the null hypothesis of independence: $\theta_{1}=0$)
- G² is log likelihood ratio test
- $\hat{\theta}$ is full model likelihood estimator
- N is the number of trials
- $n_{i}$ is the number of individuals in trial i

In other words, the mutual information can be estimated performing the log likelihood ratio test between models **Equation D** and **Equation E**. The $R_{\mathrm{hindiv}}^{2}$ and the LRF can be estimated based on the MI estimate.

Note that while $R_{\mathrm{hindiv}}^{2}$ ​can be derived using models that use one single time point, more complex settings can also be handled. The LRF handles longitudinal models with repeatedly measured SEPs and CEPs over time (See **Equation D** and **Equation E**, see details in the main paper, how these models have been handled). Asymptotic confidence intervals for $R_{\mathrm{hindiv}}^{2}$ and LRF can be estimated following the approach of Kent and Alonso ^5^ ^6^.

## **Example: Quantifying information shared between two binary variables using mutual information**

In this section, we determine the Mutual Information (MI) between two binary random variables as well as a score and a binary outcome (typically related by a logistic regression model).

1. **Mutual information derived from 2 by 2 contingency table**

To quantify the statistical dependence between two binary random variables - here labeled as SEP (surrogate endpoint) and CEP (clinical endpoint) - we use the concept of Mutual Information (MI). This method stems from information theory and captures the reduction in uncertainty of one variable given knowledge of the other. Therefore, MI is a useful concept in surrogate evaluation procedures, as it quantifies the information gain about the CEP provided by the SEP.

Let us consider the following 2 × 2 contingency table representing joint frequencies of SEP and CEP:

|  | **CEP = 0** | **CEP = 1** |
| --- | --- | --- |
| **SEP = 0** | 1000 | 500 |
| **SEP = 1** | 500 | 1000 |

The Shannon entropy ($H$) of a discrete variable is a measure of its uncertainty. For CEP, the entropy is defined as:

**Equation H: Calculation of the Shannon entropy of binary random variables**

$$H\left( \mathrm{CEP} \right)=-\sum P\left( CEP=c \right)\cdot log\left( P\left( CEP=c \right) \right),$$

where $P\left( \mathrm{CEP} \right)$is the probability of observing $CEP = c$ and $\log$ is the natural logarithm.

We computed the marginal probabilities of the CEP using the contingency table:

$$P\left( CEP=0 \right)=0.5,$$

$$P\left( CEP=1 \right)=0.5$$

$$H\left( \mathrm{CEP} \right) = -\left[ 0.5 \cdot log\left( 0.5 \right) + 0.5 \cdot log\left( 0.5 \right) \right]=0.6931472 Nats,$$

where $\log$ is the natural logarithm.

Conditional entropy $H\left( \mathrm{CEP} | \mathrm{SEP} \right)$ measures the remaining uncertainty of the CEP after observing the SEP. It is defined as:

**Equation I: Calculation of conditional Shannon entropy of two binary random variables**

$$H\left( CEP|SEP \right)=\sum P\left( SEP=s \right)\cdot\left( -\sum P\left( CEP=c|SEP=s \right)\cdot log P\left( CEP= c|SEP=s \right) \right) = 0.6365142 Nats$$

where:

- $s$indexes the values of SEP (0 and 1)
- $c$indexes the values of CEP (0 and 1)
- $\log$ is the natural logarithm

The mutual information $\mathrm{MI}\left( CEP;SEP \right)$is the reduction in entropy of CEP when SEP becomes known:

**Equation J: Estimation of the Mutual Information (MI)**

$$MI\left( CEP,SEP \right)=H\left( CEP \right)-H\left( CEP | SEP \right)=$$

$$0.6931472Nats-0.6365142 Nats=0.05233633Nats$$

Thus, knowing SEP reduces the uncertainty in CEP by 0.05233633Nats.

An equivalent approach to calculating MI is:

$$MI = \sum P\left( SEP=s, CEP=c \right)\cdot log\left[ P\left( CEP= c, SEP=s \right)/\left( P\left( SEP=s \right)P\left( CEP=c \right) \right) \right] = 0.05233633\mathrm{Nats}$$

To scale MI to a [0,1] range analogous to an $R^{2}$measure, we use $R_{\mathrm{bindiv}}^{2}$ (an equivalent to LRF, when SEP and CEP are binary and measured only once) as presented in chapter 10.6 by Alonso and colleagues ^7^:

**Equation K: Estimation of** $R_{bindiv}^{2}$

$$R_{bindiv}^{2}\frac{MI\left( CEP,SEP \right)}{min\left( H\left( CEP \right),H\left( SEP \right) \right)},$$

with:

$$H\left( \mathrm{SEP} \right) = -\left[ 0.5 \cdot log\left( 0.5 \right) + 0.5 \cdot log\left( 0.5 \right) \right]=0.6931472 Nats,$$

where $\log$ is the natural logarithm.

Hence:

$$R_{\mathrm{bindiv}}^{2}=\frac{0.036196039Nats}{0.6931472Nats}=0.05233633$$

Examples and R script are provided in the https://osf.io (https://osf.io/ht4su/overview).

1. **Mutual information derived from logistic regression models**

An alternative approach to estimate MI is the usage of the log-likelihoods from (logistic) regression models. This method proceeds as follows:

1. Fit a null model predicting CEP with only an intercept:

$f\left( \mathrm{CEP} \right): CEP_{j}=\mu_{c}+\epsilon_{\mathrm{Cj}}$

2. Fit a null model for SEP:

$f\left( \mathrm{SEP} \right): SEP_{j}=\mu_{S}+\epsilon_{\mathrm{Sj}}$

3. Fit a full model predicting CEP using SEP:

$f(CEP|SEP): CEP|SEP_{j}=\mu+\beta SEP_{j}+\epsilon_{j}$,

where:

- $j$= 1, 2, …, n subjects
- $\mathrm{SEP}$: Surrogate endpoint
- $\mathrm{CEP}$: Clinical endpoint
- $\mu,\mu_{s},\mu_{c}$: Model specific intercepts
- $\beta$ the coefficient of the $\mathrm{SEP}$

4. Compute the entropies using the negative log-likelihood per observation:
 $H\left( \text{CEP} \right)=-\frac{\log L\left( f\left( \mathrm{CEP} \right) \right)}{N} = 0.6931472\mathrm{Nats}$
 $H\left( \text{SEP} \right)=-\frac{\log L\left( f\left( \mathrm{SEP} \right) \right)}{N} = 0.6931472 Nats$
 $H\left( \text{SEP|CEP} \right)=-\frac{\log L\left( f\left( \mathrm{CEP} | \mathrm{SEP} \right) \right)}{N} = 6365142 Nats$,

where:

- $\mathrm{SEP}$: Surrogate endpoint
- $\mathrm{CEP}$: Clinical endpoint
- $f\left( \mathrm{SEP} \right)$*,* $f\left( \mathrm{CEP} \right)$*,* and $f\left( \mathrm{CEP} | \mathrm{SEP} \right)$ are density functions of $\mathrm{CEP}$, $\mathrm{SEP}$, and $CEP|SEP$estimated by a generalized linear model
- $N$: sample size
- $H$: Shannon entropy
- $\log$: natural logarithm
- $L$: log likelihood of a model

MI and $R_{\mathrm{bindiv}}^{2}$ were estimated as defined in ***Equation J*** and ***Equation K*** in the previous subsection **3a)**. Estimating Shannon entropies using generalized linear models provides the opportunity to generalize the calculation of MI, allowing for flexible handling of the underlying distributions of potential SEPs and CEPs (see chapters 9 and 10 in the book of Alonso ^7^.). Section **2,** for example, illustrates how to estimate $R_{\mathrm{hindiv}}^{2}$ which is based on continuous SEP and CEP measures, or the LRF, which can incorporate repeated measurements over time.

To illustrate the behavior of the LRF in relation to odds ratios (ORs), we simulated a binary outcome Y (representing the CEP) as a function of three binary predictors X1, X2, X3 (representing components of a SEP). The data-generating mechanism was based on a logistic regression model:

$$logit\left( P\left( Y=1 \right) \right)=-1+1.2\cdot X1+0.8\cdot X2+1.5\cdot X3$$

We simulated data for $n = 1000$ individuals using independent Bernoulli distributions: $X1\sim Bernoulli\left( 0.5 \right), X2\sim Bernoulli\left( 0.4 \right),and X3\sim Bernoulli\left( 0.3 \right)$. The binary outcome was generated according to the model above.

Fitting a logistic regression to the simulated data, yielded the following coefficient estimates:

$$\hat{\beta_{1}}=1.140\quad\left( \text{O}\text{R}_{\text{X}_{\text{1}}}=e^{1.140}=3.13, p=2.86\times{10}^{-15} \right)$$

$$\hat{\beta_{2}}=0.810\quad\left( \text{O}\text{R}_{\text{X}_{\text{2}}}=e^{0.810}=2.25,p=5.86\times{10}^{-8} \right)$$

$$\hat{\beta_{3}}=1.625\quad\left( \text{O}\text{R}_{\text{X}_{\text{3}}}=e^{1.625}=5.08,p<2\times{10}^{-16} \right)$$

These results indicate statistically significant and may be interpreted as clinically meaningful associations between each SEP component and the CEP.

To assess the informativeness of the SEP (joint distribution of X1, X2, and X3) for predicting the CEP (= Y) from an information-theoretic perspective, we computed the mutual information (MI) between the binary CEP and the joint distribution of X1, X2, and X3 (= SEP)​ grouped by their unique combinations ("risk groups"). The mutual information was calculated using:

$$MI\left( CEP,\text{SEP} \right)=\sum_{SEP,y} p\left( SEP,CEP \right)\log_{2} \left( \frac{p\left( SEP,CEP \right)}{p\left( SEP \right) p\left( CEP \right)} \right)$$

Inserting the empirical probabilities from the grouped data into the formula yielded:

$$MI\left( Y,\text{SEP} \right)=0.136$$

Based on this, the Likelihood Reduction Factor (LRF) was computed as:

$$\text{LRF}=1-\exp\left( -2\cdot MI\left( Y,\text{SEP} \right) \right)=1-\exp\left( -2\cdot0.136 \right)=0.234$$

This example highlights a key insight: although the ORs are large and statistically highly significant, the LRF, reflecting the amount of shared information between the CEP and the SEP, is comparatively small. In this case, LRF = 0.234 meaning that only about 23.4% of the variance in the clinical outcome is explained bey the SEP (X1, X2, and X3). This underscores that even strong average associations may not imply high individual-level predictability and that information-based metrics such as MI and LRF provide complementary perspectives in the evaluation of SEPs and in general prognostic factors.

The R-code to reproduce this examples is on https://osf.io (https://osf.io/ht4su/overview).

1. **Application of mutual information to derive the likelihood reduction factor in individual level surrogacy evaluation**

Here we explain the relevance of LRF and its relationship to MI represented by the following formula:

$LRF= 1-e^{-2\cdot MI\left( SEP,CEP \right)}=1-e^{- \frac{G^{2}}{n}}$.

To derive the likelihood reduction factor (LRF) one applies the information theoretic (IT) approach introduced by Alonso and colleagues ^7^. See also previous subsections **3a)** and **3b)** and section **2**. The LRF is a function of MI (see **Equation G**), which measures shared information content of two random variables ^7,8^. The central idea is to treat SEP and CEP as random variables and to measure how much information the SEP provides about a CEP.

To generalize the estimation of the MI for evaluating SEPs and CEPs regardless of their underlying distributions, $H\left( \mathrm{CEP} | \mathrm{SEP} \right)-H\left( \mathrm{CEP} \right)$ can be interpreted as the mean individual contribution of the log-likelihood difference between a full statistical regression model that includes a treatment indicator Z and the SEP, and a reduced model excluding the SEP: $\mathrm{MI}\left( X,Y \right)=\frac{1}{n}\sum\left( \log\left[ f\left( \mathrm{CEP}_{i} | SEPi,\hat{\theta} \right) \right]-log\left[ f\left( \mathrm{CEP}_{i} | \hat{\theta_{0}} \right) \right] \right)=\frac{1}{n}G^{2}$, where $f$ denotes density functions, $\hat{\theta}$ and $\hat{\theta_{0}}$ are the maximum likelihood estimates of the model parameters for the full (including the SEP) respective the reduced (excluding the SEP) model, $n$ is the number of individuals, and $G^{2}$is the likelihood ratio test statistic. The LRF (the metric for ILS evaluation of repeatedly measured SEP and CEP) can then be estimated as $LRF= 1-e^{-2\cdot MI\left( SEP,CEP \right)}=1-e^{- \frac{G^{2}}{n}}$. From this perspective, MI and the derived LRF quantify how much information content of the SEP reduces uncertainty of the prediction of the CEP by measuring the increase in likelihood (**Figure C** in section **4).** Hence, the LRF is conceptually similar to the $R^{2}$ statistic, as it reflects the proportion of explained variability. Unlike parameter-dependent measures, it does not rely on specific distributional assumptions. Therefore, the IT approach offers several advantages: (1) it does not require a joint model between SEP and CEP to quantify their association, (2) it accommodates non-linear and complex relationships, (3) it can handle continuous, categorical, and other outcome types, and (4) it allows for non-parametric estimation.

## **PTE, Likelihood ratio test, and** $\mathbf{R}_{\mathbf{hindiv}}^{\mathbf{2}}$**: an illustration of group-based versus individual-level, variance-reduction-based surrogacy metrics.**

This section demonstrates the limitations of group-level based prediction evaluation metrics such as the PTE (which is based on model coefficients - see section **1.** for details) or the log-likelihood ratio (LR) test to assess the predictive ability performance of prognostic factors like SEPs. We present an intuitive example that illustrates three key observations across varying levels of prediction accuracy. First, PTE values remain consistently high. Second, p-values from the LR test comparing the models in Equation D and Equation E remain consistently low. This is against our expectations as both metrics show similar values even when the prediction of the CEP by the SEP becomes increasingly imprecise. Hence, we refer to measures such as mutual information (MI), which underlie $R_{\mathrm{hindiv}}^{2}$​, to which the LRF reduces in settings where both the SEP and CEP are not measured longitudinally. These measures capture prediction accuracy through variance reduction. See Sections **2** and **3** for methodological details.

**Data Simulation**
For each combination of variance in the SEP and CEP, data were simulated for two study arms (treated and untreated), each with 1000 individuals:

$$\mathrm{SE}P_{i}^{\mathrm{untreated}} \sim N\left( \mu=6,\sigma= 0.5 \right); and SEP_{i}^{\mathrm{treated}} \sim N\left( \mu=4,\sigma=0.5,1,\ldots,2 \right)$$

$$\mathrm{CE}P_{i}^{\mathrm{untreated}}=\alpha\cdot\mathrm{SEP}_{i}^{\mathrm{untreated}}+\epsilon_{i}; and CEP_{i}^{\mathrm{treated}}=\alpha\cdot\mathrm{SEP}_{i}^{\mathrm{treated}}+\epsilon_{i},$$

where:

- $\mathrm{SEP}$ = surrogate endpoint
- $\mathrm{CEP}$ = clinical endpoint
- $i=1,2,\ldots,n_{p}$*,* with $n_{p}$ = 1000
- $N\left( \mu,\sigma\right)$ represents a standard normal distribution with mean $\mu$ and standard deviation $\sigma$
- $\epsilon_{i}\sim N\left( \mu=0,\sigma=0.5,1,1.5,\ldots,10 \right)$
- $\alpha= 1.5$ induces the grade of association between $\mathrm{SEP}$ and $\mathrm{CEP}$


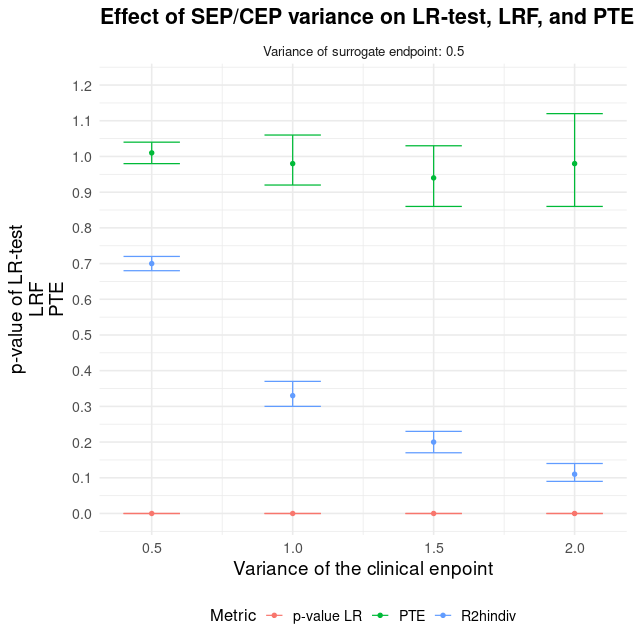


**Figure C** shows the results of the simulation introduced in this section, when $\alpha= 1.5$ (the code is provided on https://osf.io (https://osf.io/ht4su/overview). Despite the PTE consistently yielding high values around one, $R_{\mathrm{hindiv}}^{2}$ (equivalent to LRF) decreases with increasing uncertainty in the CEP. This illustrates that the PET does not consider the variance reduction of a future CEP by predictive factors such as SEPs. However, considering variance reduction is essential for generating accurate individual predictions of future CEPs, which are crucial for informed treatment decisions. In addition, in certain settings, the association between SEP and CEP is overestimated by PET. A similar pattern is observed when studying the p-values of the LR test. Again, despite decreasing values of $R_{\mathrm{hindiv}}^{2}$ the p-values of the log-likelihood ratio (LR) test remain near zero, indicating significant improvements of the full model (including the SEP) over the reduced model (excluding the SEP). Hence, significant improvements in model performance as measured by group-based changes in log-likelihood between two models may lead to the conclusion that statistical significance alone is sufficient evidence for the impact of a prognostic factor on a future CEP. Obviously, this conclusion does not necessarily hold as predictive accuracy may still be low as evidenced by low values of $R_{\mathrm{hindiv}}^{2}$​ despite the presence of statistically significant LR tests.


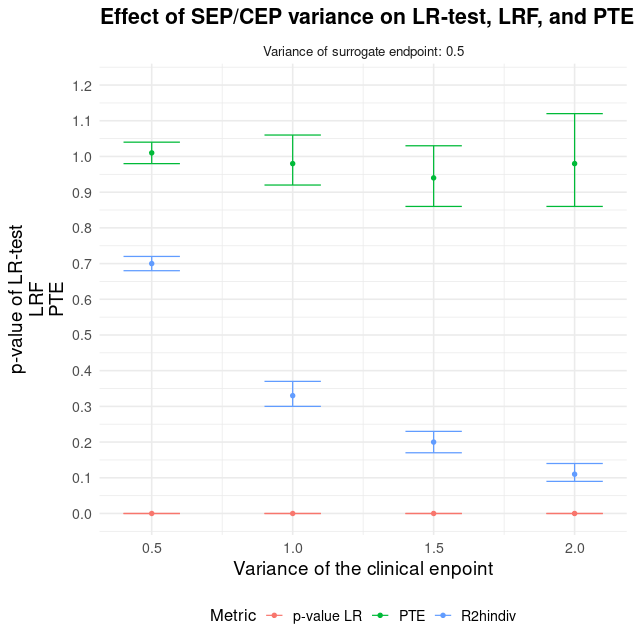


**Figure C*:*** Effect of SEP/CEP variance on several prediction performance metrics (when SEP/CEP inducing association $\alpha=1.5$)

Effect of increasing the variance of the clinical endpoint (CEP) by constant surrogate endpoint (SEP) variance of 0.5 on three different metrics, namely proportion of treatment effect explained (PTE), R²hindiv, and p-value from log-Likelihood-Ratio (LR) test. Error bars represent 95% confidence intervals based on 1000 bootstrap iterations. The strength of association between SEP and CEP was fixed at $\alpha= 1.5$. The code is provided on https://osf.io (https://osf.io/ht4su/overview).

## **Relationship between positive/negative predictive values considering SEP/CEP prevalence.**

The literature does not seem to report clear metrics of predictive performance of predictive tools in the context of multiple sclerosis (MS). For example, the 2015 MAGNIMS recommendation paper provides mostly sensitivity and specificity values in Table 1 ^9^. However, to accurately assess the predictive performance of a prediction tool, the prevalence of both the prognostic factor of interest and the CEP must be considered. In general, the prevalence is incorporated in metrics such as the positive and negative predictive values (PPV and NPV). In the context of MS, the prevalence of new or newly enlarged T2 lesions and/or relapses may be particularly relevant.

In **Figure *D***, PPVs and NPVs are presented across a range of prevalences from zero to one. The sensitivity and specificity values used for this calculation are taken from Table 1 of the 2015 MAGNIMS paper, which reported several prediction tools in MS ^9^. **Figure *D*** illustrates increasing PPV (decreasing NPV) values with rising prevalence, emphasizing that even relatively high sensitivity and/or specificity values alone are insufficient to evaluate the predictive performance of a tool intended to inform clinical decision-making. Therefore, in the context of MS, the PPV and NPV incorporating the prevalence of T2 lesions or new relapses should be applied when evaluating the predictive power of a prediction tool.

The Likelihood Reduction Factor (LRF), in contrast to the PPV, is an unconditional measure. It considers the entire data distribution, not just the subset of true and false positives as the PPV (= $\frac{\text{Sensitivity }\cdot\text{Prevalence}}{\text{Sensitivity }\cdot\text{Prevalence }+\left( 1-\text{Specificity} \right)\cdot\left( 1-\text{Prevalence} \right)}$ = $\frac{TP}{TP+FP}$does. To demonstrate the behavior of the LRF under various conditions, we now perform step-by-step calculations based on its formal definition. The LRF is defined as:

$$LRF=1-\exp\left( -2\cdot MI\left( SEP,CEP \right) \right)$$

where the mutual information (MI) between a surrogate endpoint (SEP) and a clinical endpoint (CEP) is given by:

$$MI\left( SEP,CEP \right)=H\left( CEP \right)-H\left( CEP \mid SEP \right)$$

The Shannon entropy of a binary variable X with success probability p is defined as:

$$H\left( X \right)=-p\cdot\log_{2} \left( p \right)-\left( 1-p \right)\cdot\log_{2} \left( 1-p \right)$$

**Case 1: High Sensitivity and Specificity (0.99) with SEP Prevalence = 0.5**

Assume the following values:
- Sensitivity = 0.99 → P(CEP = 1 | SEP = 1) = 0.99
- Specificity = 0.99 → P(CEP = 0 | SEP = 0) = 0.99
- SEP prevalence = 0.5 → P(SEP = 1) = 0.5, P(SEP = 0) = 0.5

$$P\left( CEP=1 \right)=0.5\cdot0.99+0.5\cdot0.01=0.5$$

$$P\left( CEP=0 \right)=1-0.5=0.5$$

$$H\left( CEP \right)=-0.5\cdot\log_{2} \left( 0.5 \right)-0.5\cdot\log_{2} \left( 0.5 \right)=1$$

$$H\left( CEP \mid SEP=1 \right)=-0.99\cdot\log_{2} \left( 0.99 \right)-0.01\cdot\log_{2} \left( 0.01 \right)\approx0.0808$$

$$H\left( CEP \mid SEP=0 \right)=-0.01\cdot\log_{2} \left( 0.01 \right)-0.99\cdot\log_{2} \left( 0.99 \right)\approx0.0808$$

$$H\left( CEP \mid SEP \right)=0.5\cdot0.0808+0.5\cdot0.0808=0.0808$$

$$MI=H\left( CEP \right)-H\left( CEP \mid SEP \right)=1-0.0808=0.9192\text{ bits}$$

$$MI_{nats}=0.9192\cdot\ln\left( 2 \right)\approx0.6368$$

$$LRF=1-\exp\left( -2\cdot0.6368 \right)\approx1-\exp\left( -1.2736 \right)\approx1-0.279=0.721$$

$$PPV=\frac{0.99\cdot0.5}{0.99\cdot0.5+\left( 1-0.99 \right)\cdot\left( 1-0.5 \right)}=\frac{0.495}{0.495+0.005}=\frac{0.495}{0.5}=0.99$$

**Case 2: Low Sensitivity and Specificity (0.6) with SEP Prevalence = 0.5**

$P\left( CEP=1 \right)=0.5\cdot0.6+0.5\cdot0.4$ = 0.5 → H(CEP) = 1

$$H\left( CEP \mid SEP=1 \right)=H\left( CEP \mid SEP=0 \right)=-0.6\cdot\log_{2} \left( 0.6 \right)-0.4\cdot\log_{2} \left( 0.4 \right)\approx0.97095$$

$$MI=1-0.97095=0.02905\text{ bits}$$

$$MI_{nats}=0.02905\cdot\ln\left( 2 \right)\approx0.0201$$

$$LRF=1-\exp\left( -2\cdot0.0201 \right)\approx0.0394$$

$$PPV=\frac{0.6\cdot0.5}{0.6\cdot0.5+\left( 1-0.6 \right)\cdot\left( 1-0.5 \right)}=\frac{0.3}{0.3+0.2}=\frac{0.3}{0.5}=0.6$$

**Case 3: Low Sensitivity/Specificity (0.6) with SEP Prevalence = 0.1**

$$P\left( CEP=1 \right)=0.1\cdot0.6+0.9\cdot0.4=0.42$$

$$H\left( CEP \right)=-0.42\cdot\log_{2} \left( 0.42 \right)-0.58\cdot\log_{2} \left( 0.58 \right)\approx0.9852$$

$$H\left( CEP \mid SEP \right)=0.97095$$

$$MI=0.9852-0.97095=0.01425\text{ bits}$$

$$MI_{nats}=0.01425\cdot\ln\left( 2 \right)\approx0.00987$$

$$LRF=1-\exp\left( -2\cdot0.00987 \right)\approx0.0196$$

$$PPV=\frac{0.6\cdot0.1}{0.6\cdot0.1+\left( 1-0.6 \right)\cdot\left( 1-0.1 \right)}=\frac{0.06}{0.06+0.36}=\frac{0.06}{0.42}\approx0.143$$

**Case 4: Low Sensitivity/Specificity (0.6) with SEP Prevalence = 0.9**

$$P\left( CEP=1 \right)=0.9\cdot0.6+0.1\cdot0.4=0.58$$

$$H\left( CEP \right)=-0.58\cdot\log_{2} \left( 0.58 \right)-0.42\cdot\log_{2} \left( 0.42 \right)\approx0.9852$$

$$MI=0.9852-0.97095=0.01425\text{ bits}$$

$$MI_{nats}=0.01425\cdot\ln\left( 2 \right)\approx0.00987$$

$$LRF=1-\exp\left( -2\cdot0.00987 \right)\approx0.0196$$

$$PPV=\frac{0.6\cdot0.9}{0.6\cdot0.9+\left( 1-0.6 \right)\cdot\left( 1-0.9 \right)}=\frac{0.54}{0.54+0.04}=\frac{0.54}{0.58}\approx0.931$$

***Table B:*** *Summary of shown scenarios*

| **Scenario** | **Sensitivity** | **Specificity** | **Prevalence (SEP)** | **PPV** | **LRF** |
| --- | --- | --- | --- | --- | --- |
| 1 | 0.99 | 0.99 | 0.5 | 0.990 | 0.721 |
| 2 | 0.60 | 0.60 | 0.5 | 0.600 | ≈ 0.039 |
| 3 | 0.60 | 0.60 | 0.1 | ≈ 0.143 | ≈ 0.020 |
| 4 | 0.60 | 0.60 | 0.9 | ≈ 0.931 | ≈ 0.020 |


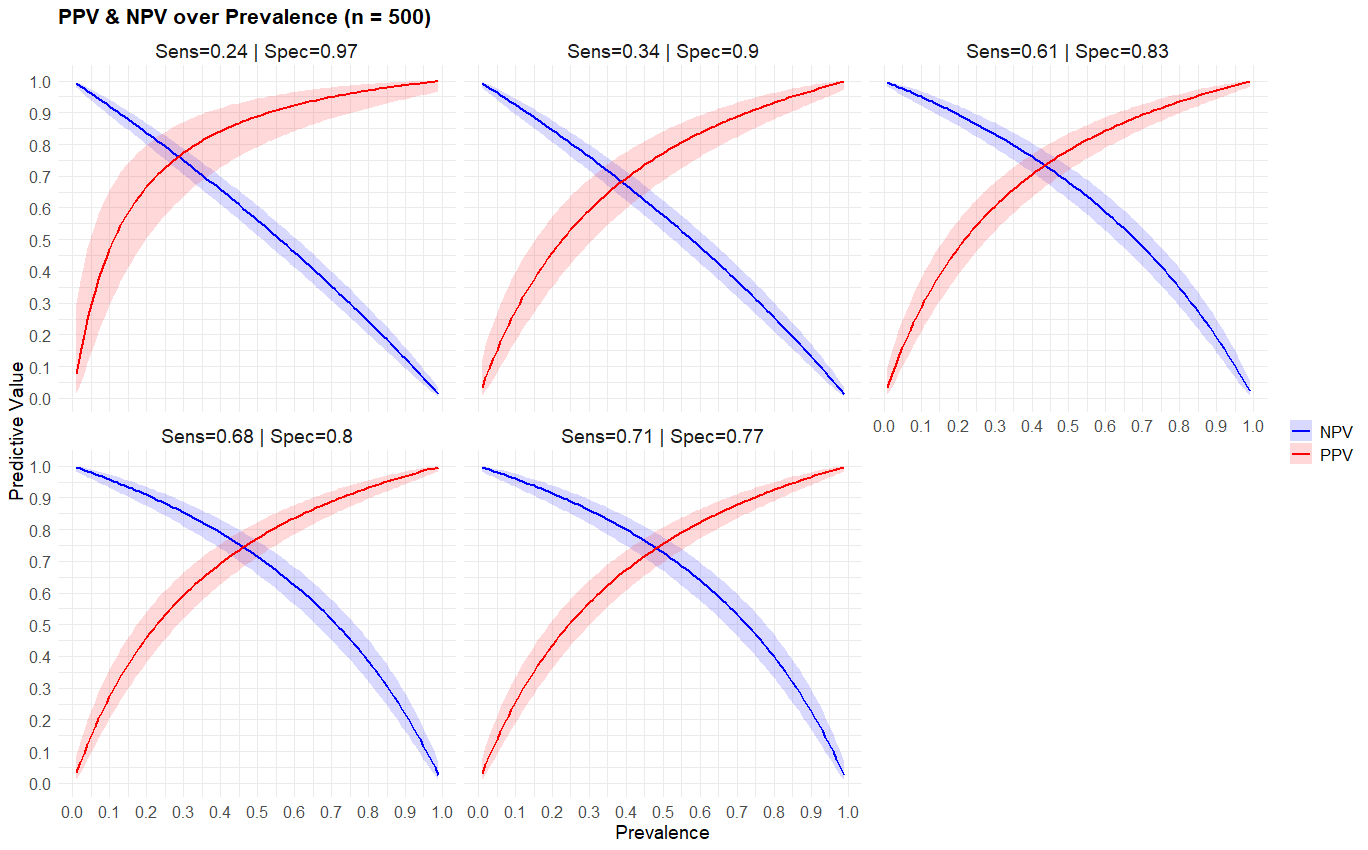


**Figure D:** Positive Predictive Value and Negative Predictive Value considering different prevalences

The Positive Predictive Value (PPV) and the Negative Predictive Value (NPV) as a function of the prevalence, calculated for five different combinations of sensitivity and specificity derived from the MAGNIMS 2015 recommendation paper (Table 1 of MAGNIMS 2015 paper) 10. Each panel illustrates how PPV (red) increases and NPV (blue) decreases with rising prevalence, based on fixed sensitivity (Sens) and specificity (Spec) values. Shaded areas represent confidence intervals. The code is provided on https://osf.io (https://osf.io/ht4su/overview).

## **References**

1. Prentice RL. Surrogate endpoints in clinical trials: Definition and operational criteria. *Statistics in medicine*. 1989;8(4):431-440.

2. Freedman LS, Graubard BI, Schatzkin A. Statistical validation of intermediate endpoints for chronic diseases. *Statistics in medicine*. 1992;11(2):167-178.

3. Sormani M, Stubinski B, Cornelisse P, Rocak S, Li D, Stefano ND. Magnetic resonance active lesions as individual-level surrogate for relapses in multiple sclerosis. *Multiple Sclerosis Journal*. 2011;17(5):541-549.

4. Alonso A, Molenberghs G. Surrogate marker evaluation from an information theory perspective. *Biometrics*. 2007;63(1):180-186.

5. Alonso A, Molenberghs G, Geys H, Buyse M, Vangeneugden T. A unifying approach for surrogate marker validation based on Prentice’s criteria. *Statistics in medicine*. 2006;25(2):205-221.

6. Kent JT. Information gain and a general measure of correlation. *Biometrika*. 1983;70(1):163-173.

7. Alonso A, Bigirumurame T, Burzykowski T, et al. *Applied Surrogate Endpoint Evaluation Methods with Sas and r*. CRC Press; 2016.

8. Ensor H, Weir CJ. Evaluation of surrogacy in the multi-trial setting based on information theory: An extension to ordinal outcomes. *Journal of Biopharmaceutical Statistics*. 2020;30(2):364-376. doi:10.1080/10543406.2019.1696357

9. Wattjes MP, Rovira À, Miller D, et al. MAGNIMS consensus guidelines on the use of MRI in multiple sclerosis--establishing disease prognosis and monitoring patients. *Nature Reviews Neurology*. 2015;11(10):597-607.
