# Supplementary material for: Individual-level surrogacy of MRI lesions for disease severity in RRMS: Methods to quantify predictive power and their application to longitudinal data from recent trials
Source: PLoS One. 2025 Dec 26;20(12):e0337893. doi: 10.1371/journal.pone.0337893 (PMC12742783; doi:10.1371/journal.pone.0337893)
Supplement: S2 Text — (DOCX) [file pone.0337893.s002.docx]

**S2 text: Simulation study**

At a first step, the SEP was sampled for $k$ time points from a multivariate normal distribution with mean zero and considering $k x k$correlation matrix $R_{S}$ with a first order auto-regressive autocorrelation with $\phi= 0.8$:

$\mathrm{SE}P_{\mathrm{ik}}\sim MNV\left( \mu,\Sigma_{\mathrm{SS}} \right)$ *,*

*with:*

- $i=1,2,\ldots,n_{p}$
- $n_{p}=number of patients \left( 100, 300 or 600 \right)$
- $\mu= 0$
- $\Sigma_{S}=D*R_{S}* D$
- $D=1_{k x k}*$ $\epsilon$
- $\epsilon= 1$

$\Sigma_{S}$ was adjusted according to the methodology of Barbiero and Ferrari ^1^. CEP was estimated afterwards:

$\mathrm{CE}P_{\mathrm{ik}}\sim MNV\left( \alpha*SEP_{\mathrm{ik}},\Sigma_{\mathrm{SS}} \right)$*,*

*with* $\alpha= 0.1, 0.5, or 2.25$

Starting from $\mathrm{CE}P_{\mathrm{ik}}$and $\mathrm{SE}P_{\mathrm{ik}}$, Poisson distributed $\mathrm{CE}P_{\mathrm{ik}}^{\mathrm{Poisson}}$and $\mathrm{SE}P_{\mathrm{ik}}^{\mathrm{Poisson}}$were generated:

- $\mathrm{SE}P_{\mathrm{ik}}^{\mathrm{Poisson}} \sim Pois_{\mathrm{cum}}^{-1}\left( p_{\mathrm{SE}P_{i}},\lambda\right)$
- $\mathrm{CE}P_{\mathrm{ik}}^{\mathrm{Poisson}} \sim Pois_{\mathrm{cum}}^{-1}\left( p_{\mathrm{CE}P_{i}},\lambda\right)$*, with*
- $p_{\mathrm{SE}P_{i}} = P_{\mathrm{norm}}\left( \mathrm{SE}P_{\mathrm{ik}},\mu, \epsilon\right)$
- $p_{\mathrm{CE}P_{i}} = P_{\mathrm{norm}}\left( \mathrm{CE}P_{\mathrm{ik}},\mu, \epsilon\right)$
- $\lambda= 2$ *, and where*
- $\mathrm{Poi}s_{\mathrm{cum}}^{-1} is the inverse Poisson cumulative density function$
- $P_{\mathrm{norm}} is the normal probability function$

Additionally. Starting from $\mathrm{SE}P_{\mathrm{ik}}^{\mathrm{Poisson}}$and $\mathrm{CE}P_{\mathrm{ik}}^{\mathrm{Poisson}}$**,** the transformation algorithm by Huber and colleagues were applied to generate normally distributed data ^2^. Finally, 18 data sets with 2 or 4 measurement timepoints, 100, 300, or 600 subjects, and three different values for $\alpha$ (0.1, 0.5, 2.25) were generated. Each data set includes correlated normally distributed, Poisson distributed, and transformed SEP and CEP. In this way, all SEP/CEP combinations can be created (Gaussian – Gaussian, Gaussian – Poisson, Poisson – Gaussian, Poisson – Poisson, Gaussian – transformed Poisson, transformed Poisson – Gaussian, and transformed Poisson - transformed Poisson)

The true LRF or $R_{\Lambda}^{2}$ is calculated as follows:

A $k x k$correlation matrix $R_{S}$ with a first order auto-regressive autocorrelation with $\phi= 0.8$ and $k$ representing the number of measurement time points were defined to calculate LRF and $R_{\Lambda}^{2}$*:*

$R_{\Lambda}^{2}= LRF = \left( 1-\Lambda\right)$*,*

*where:*

- $\Lambda=\frac{\left| \Sigma\right|}{\left| \Sigma_{\mathrm{CC}} \right|\left| \Sigma_{\mathrm{SS}} \right|}$
- $\Sigma=$ $\left[ \begin{matrix} \Sigma_{\mathrm{SS}} & \Sigma_{\mathrm{SC}} \\ \Sigma_{\mathrm{CS}} & \Sigma_{\mathrm{CC}} \end{matrix} \right]$
- $\Sigma_{\mathrm{SS}}=D*R_{S}* D$
- $\Sigma_{\mathrm{SC}}= \Sigma_{\mathrm{CS}} =\alpha* \Sigma_{\mathrm{SS}}$
- $\Sigma_{\mathrm{CC}}=\left( \alpha^{2}+\epsilon^{2} \right)*\Sigma_{\mathrm{SS}}$
- $D=1_{k x k}*$ $\epsilon$
- $\epsilon= 1$
- $\alpha= 0.1, 0.5, or 2.25$

The script to generate the simulated data can be found on https://osf.io (https://osf.io/ht4su/overview).

1. Barbiero A, Ferrari PA. Simulation of correlated Poisson variables. *Applied Stochastic Models in Business and Industry*. 2015;31(5):669-680.

2. Huber W, Von Heydebreck A, Sültmann H, Poustka A, Vingron M. Variance stabilization applied to microarray data calibration and to the quantification of differential expression. *Bioinformatics*. 2002;18(suppl_1):S96-S104.
