# Supplementary material for: Individual-level surrogacy of MRI lesions for disease severity in RRMS: Methods to quantify predictive power and their application to longitudinal data from recent trials
Source: PLoS One. 2025 Dec 26;20(12):e0337893. doi: 10.1371/journal.pone.0337893 (PMC12742783; doi:10.1371/journal.pone.0337893)
Supplement: S3 Text — (DOCX) [file pone.0337893.s003.docx]

**S3 text: List of R-packages**

- *sas7bdat 0.6*
- *tidyverse 1.3.2*
- *haven 2.5.1*
- *lubridate 1.9.2*
- *rlist 0.4.6.2*
- *MASS 7.3.58.1*
- *lme4 1.1.32*
- *pbapply 1.7.0*
- *parallel 4.2.2*
- *nlme 3.1.162*
- *ggcorrplot 0.1.4*
- *glmmTMB 1.1.6*
- *ordinal 2022.11.16*
- *foreach 1.5.2*
- *doParallel 1.0.17*
- *brms 2.18.0*
- *cAIC4 1.0*
- *mgcv 1.8.41*
- *boot 1.3.28*
- *MCMCpack 1.6.3*
- *Matrix 1.5.3*
- *mvtnorm 1.1.3*
- *GenOrd 1.4.0*
- *data.table 1.14.6*
- *ggh4x 0.2.5*
- *rstan 2.26.15*
- *ggarchery 0.4.2*
- *kableExtra 1.3.4*
- *grid 4.2.2*
- *gridExtra 2.3*
- *ggrepel 0.9.4*
- *ggpubr 0.6.*
