# Supplementary material for: Individual-level surrogacy of MRI lesions for disease severity in RRMS: Methods to quantify predictive power and their application to longitudinal data from recent trials
Source: PLoS One. 2025 Dec 26;20(12):e0337893. doi: 10.1371/journal.pone.0337893 (PMC12742783; doi:10.1371/journal.pone.0337893)
Supplement: S1 Table — Abbreviations: RCT, randomized clinical trial; PTE, proportion of treatment effect explained; OR, odds ratio; HR, hazard ratio; EDSS, expended disability status scale; ILS, individual-level surrogacy; TRiT, treatment response in treated; TRiuT, treatment response in untreated; T2L number of new/newly enlarged T2 lesion; MAGNIMS, magnetic resonance imaging in multiple sclerosis; CMSWG, the Canadian MS Working Group; CEL, contrast enhancing lesions; TLS, trial-level surrogacy; SEP, surrogate endpoint; DMT, disease modifying treatment. (DOCX) [file pone.0337893.s004.docx]

**Table S1:** Summary of literature on T2 lesions as treatment response factor or surrogate endpoint in patients with relapsing-remitting multiple sclerosis

Abbreviations: RCT, randomized clinical trial; PTE, proportion of treatment effect explained; OR, odds ratio; HR, hazard ratio; EDSS, expended disability status scale; ILS, individual-level surrogacy; TRiT, treatment response in treated; TRiuT, treatment response in untreated; T2L number of new/newly enlarged T2 lesion; MAGNIMS, magnetic resonance imaging in multiple sclerosis; CMSWG, the Canadian MS Working Group; CEL, contrast enhancing lesions; TLS, trial-level surrogacy; SEP, surrogate endpoint; DMT, disease modifying treatment

| **Study** | **Population** | **Duration** | **Treatment** | **Association metric** | **Surrogacy** | **Association** | **Cited by** | **Comment** |
| --- | --- | --- | --- | --- | --- | --- | --- | --- |
| (Sormani, Stubinski, et al., 2011) | 1 RCT (N = 502; PRISM study) | 24 – 36 months | Interferon derivate | PTE = 0.53 [0.28 – 1.01])  PTE = 0.8 (0.34 – 1.86) | criticized ILS | T2L -> relapses | MAGNIMS 2015 | PTE is criticized |
| (Sormani, Li, et al., 2011) | 1 RCT (N = 560; PRISM study) | 24 months | Interferon derivate | PTE = 0.63 [0.2 – 2.15]  PTE = 1.00 [0.32 – 3.11] | criticized ILS | T2L –> EDSS;  T2L + relapses -> EDSS | CMSWG | PTE is criticized |
| (Río et al., 2009) | 1 Prospective study (N = 222) | 48 months | Interferon derivate | Significant ORs | TRiT | Rio score | MAGNIMS 2015 | No control; predictive ability based on group-level changes |
| (Sormani et al., 2013) | 2 RCTs (N = 373 [training cohort]; N = 222 [validation]) | 48 months | Interferon derivates | Significant HRs | TRiT | Modified Rio score | MAGNIMS 2015 | No control; predictive ability based on group-level changes |
| (Sormani et al., 2016) | 9 Clinical cohort of MAGNIMS (N = 1280) | At least 36 months | Interferon derivate | relevant changes of HRs | TRiT | MAGNIMS score | CMSWG | No control; predictive ability based on group-level changes; Unclear definition of relevant change of HRs |
| (Signori et al., 2015) | 6 RCTs (N = 6693) | Not reported | Divers DMTs | Relative treatment effect within T2L groups | TRiuT | T2L -> Annualized relapse rate | MAGNIMS 2021 | Trend of higher treatment effect within high T2 lesion load group (p = 0.068); predictive ability based on group-level changes |
| (Río et al., 2018) | 2 Clinical cohort (N = 516) | In average 6.7 years | Interferon derivate | Significant HRs | TRiT | Rio score, modified Rio score, T2L, others -> EDSS worsening | MAGNIMS 2021; CMSWG | No control; predictive ability based on group-level changes |
| (Prosperini et al., 2014) | 1 Clinical cohort (N = 370) | 48 Months | Interferon derivate | Significant HRs | TRiT | T2L/CEL -> EDSS | MAGNIMS 2015 | No control; predictive ability based on group-level changes |
| (Sormani et al., 2017) | 1 RCT (N = 552) + extension | 84 months | Teriflunomide | Significant HRs | TRiT | MAGNIMS score | CMSWG | No control; predictive ability based on group-level changes |
| (Boster et al., 2015) | 2 RCTs (N = 2355 and N = 1693) | 24 months | Fingolimod | Significant HRs | TRiT | T2L -> EDSS or relapses | CMSWG | No control; predictive ability based on group-level changes |
| (Galassi et al., 2016) | Clinical cohort (N = 392) | 60 months | Interferon derivate | Significant HRs | TRiT | T2L -> relapses | CMSWG | No control; predictive ability based on group-level changes |
| (Bermel et al., 2013) | 1 RCT/ clinical cohort (N = 136) | 15 years | Interferon derivate | Significant effect on likelihood reaching worst EDSS quartile | TRiT and TRiuT | T2L -> EDSS | CMSWG | No treatment adjustment as defined for ILS, but separate models for treatment and control arm. |

Barkhof, F., Simon, J. H., Fazekas, F., Rovaris, M., Kappos, L., De Stefano, N., Polman, C. H., Petkau, J., Radue, E. W., & Sormani, M. P. (2012). MRI monitoring of immunomodulation in relapse-onset multiple sclerosis trials. *Nature Reviews Neurology*, *8*(1), 13–21.

Bermel, R. A., You, X., Foulds, P., Hyde, R., Simon, J. H., Fisher, E., & Rudick, R. A. (2013). Predictors of long‐term outcome in multiple sclerosis patients treated with interferon beta. *Annals of Neurology*, *73*(1), 95–103. https://doi.org/10.1002/ana.23758

Boster, A., Hawker, K., Ritter, S., Tomic, D., & Sprenger, T. (2015). *Disease activity in the first year predicts longer-term clinical outcomes in the pooled population of the phase III FREEDOMS and FREEDOMS II studies (P7. 239)*.

Galassi, S., Prosperini, L., Logoteta, A., Hirsch, M. N., Fanelli, F., De Giglio, L., & Pozzilli, C. (2016). A lesion topography-based approach to predict the outcomes of patients with multiple sclerosis treated with Interferon Beta. *Multiple Sclerosis and Related Disorders*, *8*, 99–106.

Prosperini, L., Mancinelli, C. R., De Giglio, L., De Angelis, F., Barletta, V., & Pozzilli, C. (2014). Interferon beta failure predicted by EMA criteria or isolated MRI activity in multiple sclerosis. *Multiple Sclerosis Journal*, *20*(5), 566–576.

Río, J., Castilló, J., Rovira, A., Tintoré, M., Sastre-Garriga, J., Horga, A., Nos, C., Comabella, M., Aymerich, X., & Montalbán, X. (2009). Measures in the first year of therapy predict the response to interferon β in MS. *Multiple Sclerosis Journal*, *15*(7), 848–853. https://doi.org/10.1177/1352458509104591

Río, J., Rovira, À., Tintoré, M., Otero-Romero, S., Comabella, M., Vidal-Jordana, Á., Galán, I., Castilló, J., Arrambide, G., & Nos, C. (2018). Disability progression markers over 6–12 years in interferon-β-treated multiple sclerosis patients. *Multiple Sclerosis Journal*, *24*(3), 322–330.

Signori, A., Schiavetti, I., Gallo, F., & Sormani, M. (2015). Subgroups of multiple sclerosis patients with larger treatment benefits: A meta‐analysis of randomized trials. *European Journal of Neurology*, *22*(6), 960–966.

Sormani, M., Gasperini, C., Romeo, M., Rio, J., Calabrese, M., Cocco, E., Enzingher, C., Fazekas, F., Filippi, M., Gallo, A., Kappos, L., Marrosu, M. G., Martinelli, V., Prosperini, L., Rocca, M. A., Rovira, A., Sprenger, T., Stromillo, M. L., Tedeschi, G., … De Stefano, N. (2016). Assessing response to interferon-β in a multicenter dataset of patients with MS. *Neurology*, *87*(2), 134–140. https://doi.org/10.1212/WNL.0000000000002830

Sormani, M., Li, D., Bruzzi, P., Stubinski, B., Cornelisse, P., Rocak, S., & De Stefano, N. (2011). Combined MRI lesions and relapses as a surrogate for disability in multiple sclerosis. *Neurology*, *77*(18), 1684–1690.

Sormani, M., Rio, J., Tintorè, M., Signori, A., Li, D., Cornelisse, P., Stubinski, B., Stromillo, M., Montalban, X., & De Stefano, N. (2013). Scoring treatment response in patients with relapsing multiple sclerosis. *Multiple Sclerosis Journal*, *19*(5), 605–612. https://doi.org/10.1177/1352458512460605

Sormani, M., Stubinski, B., Cornelisse, P., Rocak, S., Li, D., & Stefano, N. D. (2011). Magnetic resonance active lesions as individual-level surrogate for relapses in multiple sclerosis. *Multiple Sclerosis Journal*, *17*(5), 541–549.

Sormani, M., Truffinet, P., Thangavelu, K., Rufi, P., Simonson, C., & De Stefano, N. (2017). Predicting long-term disability outcomes in patients with MS treated with teriflunomide in TEMSO. *Neurology Neuroimmunology & Neuroinflammation*, *4*(5), e379. https://doi.org/10.1212/NXI.0000000000000379
