# Supplementary material for: Individual-level surrogacy of MRI lesions for disease severity in RRMS: Methods to quantify predictive power and their application to longitudinal data from recent trials
Source: PLoS One. 2025 Dec 26;20(12):e0337893. doi: 10.1371/journal.pone.0337893 (PMC12742783; doi:10.1371/journal.pone.0337893)
Supplement: S2 Table — The table lists all combinations of surrogate endpoints (SEPs) and clinical endpoints (CEPs), each evaluated across all possible comparisons between active and control arms within of each of the used trials. Since some trials include multiple active arms, more than one comparison per trial is possible. For surrogate validation, such comparisons are essential, as both a control and a treatment arm are required to assess whether the SEP is associated with the CEP, regardless of the treatment. Trial abbreviations correspond to those used in Table 1 of the main paper. Trials marked in red provide only one of the two SEP types (either log-transformed total T2 lesion volume in cm³ or the count of new/enlarged T2 lesions). Abbreviations: EDSS, expanded disability status scale; SEP, surrogate endpoint; CEP, clinical endpoint. (DOCX) [file pone.0337893.s005.docx]

**Table S2:** Number of comparisons within trials for each SEP – CEP combination

| **Surrogate Endpoint (SEP)** | **Clinical Endpoint (CEP)** | **Trials Providing Endpoint Combination (Number of Trial Arm Comparisons)** | **Number of Comparisons between Active and Control Study Arm** |
| --- | --- | --- | --- |
| Log(T2 Volume cm³) | EDSS | CA1(2), CA2(1), CA3(2), CF2(2), CF3(2), CF4(2), WA1(1), WA2(1), WA3(4) | 17 |
| Log(T2 Volume cm³) | Number Relapses | CA1(2), CA2(1), CA3(2), CF2(2), CF3(2), CF4(2), WA1(1), WA2(1), WA3(4) | 17 |
| New/Enlarged T2 Lesions | EDSS | CA2(1), CA3(2), CF1(2), CF2(2), CF3(2), CF4(2), WA1(1), WA2(1), WA3(4) | 17 |
| New/Enlarged T2 Lesions | Number Relapses | CA2(1), CA3(2), CF1(2), CF2(2), CF3(2), CF4(2), WA1(1), WA2(1), WA3(4) | 17 |

The table lists all combinations of surrogate endpoints (SEPs) and clinical endpoints (CEPs), each evaluated across all possible comparisons between active and control arms within of each of the used trials. Since some trials include multiple active arms, more than one comparison per trial is possible. For surrogate validation, such comparisons are essential, as both a control and a treatment arm are required to assess whether the SEP is associated with the CEP, regardless of the treatment. Trial abbreviations correspond to those used in Table 1 of the main paper. Trials marked in red provide only one of the two SEP types (either log-transformed total T2 lesion volume in cm³ or the count of new/enlarged T2 lesions).

Abbreviations: EDSS, expanded disability status scale; SEP, surrogate endpoint; CEP, clinical endpoint
