# Supplementary material for: Individual-level surrogacy of MRI lesions for disease severity in RRMS: Methods to quantify predictive power and their application to longitudinal data from recent trials
Source: PLoS One. 2025 Dec 26;20(12):e0337893. doi: 10.1371/journal.pone.0337893 (PMC12742783; doi:10.1371/journal.pone.0337893)
Supplement: S3 Table — The table displays the models utilized in both the main and the sensitivity analysis for both transformed and untransformed data. It is important to note that these model strategies were applied to both simulated and clinical trial data within the amin analysis. For details about the main analysis and the sensitivity analysis please refer to the methods part of the main article. Abbreviations: GLMM, generalized linear mixed model; EDSS, expanded disability status scale; SEP, surrogate endpoint; CEP, clinical endpoint; PTE proportion of treatment effect explained. (DOCX) [file pone.0337893.s006.docx]

**Table S3:** Model strategies

The table displays the models utilized in both the main and the sensitivity analysis for both transformed and untransformed data. It is important to note that these model strategies were applied to both simulated and clinical trial data within the amin analysis. For details about the main analysis and the sensitivity analysis please refer to the methods part of the main article.

Abbreviations: GLMM, generalized linear mixed model; EDSS, expanded disability status scale; SEP, surrogate endpoint; CEP, clinical endpoint; PTE proportion of treatment effect explained

| **Surrogate Endpoint (SEP)** | **Clinical Endpoint (CEP)** | **Transformed Count Outcome** | **Model(s) Used** | **Distribution Family Used for Outcome(s)** | **Association Measurement between CEP and SEP** | **Association Metric** |
| --- | --- | --- | --- | --- | --- | --- |
| **Main Analysis: Information-Theoretic Approach (Two Models with CEP as Outcome)** | | | | | | |
| Log (T2 Volume cm³) | EDSS | no | GLMM Models | Gaussian, Ordinal | Information gained by SEP | LRF |
| Log (T2 Volume cm³) | Number Relapses | no | GLMM Models | Ordinal, Negative Binomial, Zero Inflated, Poisson | Information gained by SEP | LRF |
| New/Enlarged T2 Lesions | EDSS | no | GLMM Models | Gaussian, Ordinal | Information gained by SEP | LRF |
| New/Enlarged T2 Lesions | Number Relapses | no | GLMM Models | Ordinal, Negative Binomial, Zero Inflated, Poisson | Information gained by SEP | LRF |
| Log (T2 Volume cm³) | Number Relapses | yes | GLMM Models | Gaussian, Ordinal | Information gained by SEP | LRF |
| New/Enlarged T2 Lesions | EDSS | yes | GLMM Models | Gaussian, Ordinal | Information gained by SEP | LRF |
| New/Enlarged T2 Lesions | Number Relapses | yes | GLMM Models | Gaussian, Ordinal | Information gained by SEP | LRF |
| **Sensitivity Analysis (*longitudinal predictive*, *aggregated associative*, and *aggregated predictive* time structure setting)** | | | | | | |
| Log (T2 Volume cm³) | Number Relapses | Depending on setting aggregated | GLM (when aggregated)/GLMM (when longitudinal) | Ordinal, Negative Binomial, Zero Inflated, Poisson | Information gained by SEP | LRF |
| New/Enlarged T2 Lesions | Number Relapses | Depending on setting aggregated | GLM (when aggregated)/GLMM (when longitudinal) | Ordinal, Negative Binomial, Zero Inflated, Poisson | Information gained by SEP/ Treatment effect explained by SEP | LRF/PTE |
