# Supplementary material for: Individual-level surrogacy of MRI lesions for disease severity in RRMS: Methods to quantify predictive power and their application to longitudinal data from recent trials
Source: PLoS One. 2025 Dec 26;20(12):e0337893. doi: 10.1371/journal.pone.0337893 (PMC12742783; doi:10.1371/journal.pone.0337893)
Supplement: S5 Table — For each SEP/CEP combination dataset, the table either presents the median (and interquartile range) of EDSS or T2 volume change from baseline, derived from aggregated trial data, or includes the median and interquartile range of the relapse or new/newly enlarged T2 lesion rate per trial visit. Abbreviation: IQR, inter quartile range; SEP, surrogate endpoint; CEP, clinical endpoint. (DOCX) [file pone.0337893.s008.docx]

**Table S5*:*** Changes of endpoints over time

For each SEP/CEP combination dataset, the table either presents the median (and interquartile range) of EDSS or T2 volume change from baseline, derived from aggregated trial data, or includes the median and interquartile range of the relapse or new/newly enlarged T2 lesion rate per trial visit.

Abbreviation: IQR, inter quartile range; SEP, surrogate endpoint; CEP, clinical endpoint

| **SEP [Median (IQR)]** | **CEP [Median (IQR)]** |
| --- | --- |
| Log (T2 volume) change from baseline [-0.024 (-0.128; 0.059)] | EDSS change from baseline [0 (-0.5,0)] |
| Log (T2 volume) change from baseline [-0.031 (-0.116; 0.033)] | Relapse rate per measurement time point [0.21 (0.14,0.36)] |
| New or newly enlarged T2 lesions rate per measurement time point [1.45 (0.81;1.90)] | EDSS change from baseline [0 (0,0)] |
| New or newly enlarged T2 lesions rate per measurement time point [1.46 (0.73;2.09)] | Relapse rate per measurement time point [0.12 (0.03,0.20)] |
