# Supplementary material for: Individual-level surrogacy of MRI lesions for disease severity in RRMS: Methods to quantify predictive power and their application to longitudinal data from recent trials
Source: PLoS One. 2025 Dec 26;20(12):e0337893. doi: 10.1371/journal.pone.0337893 (PMC12742783; doi:10.1371/journal.pone.0337893)
Supplement: S1 Fig — A sensitivity analyses illustrating the individual-level surrogacy (ILS) were conducted by calculating the likelihood reduction factor (LRF) across different temporal association settings between SEP (new/enlarged T2 lesions - T2L - and T2 volume – T2V) and CEP (new relapses): longitudinal predictive (repeatedly measured SEP during the first year of the trial and repeatedly measured CEP during the second year) aggregated associative (summed-up SEP and CEP), and aggregated predictive (summed-up SEP at months 6 and 12, summed-up CEP within the second year). These analyses are restricted to data collected within the first two years of follow-up. Only trials with a duration of at least two years were included. (DOCX) [file pone.0337893.s009.docx]

**
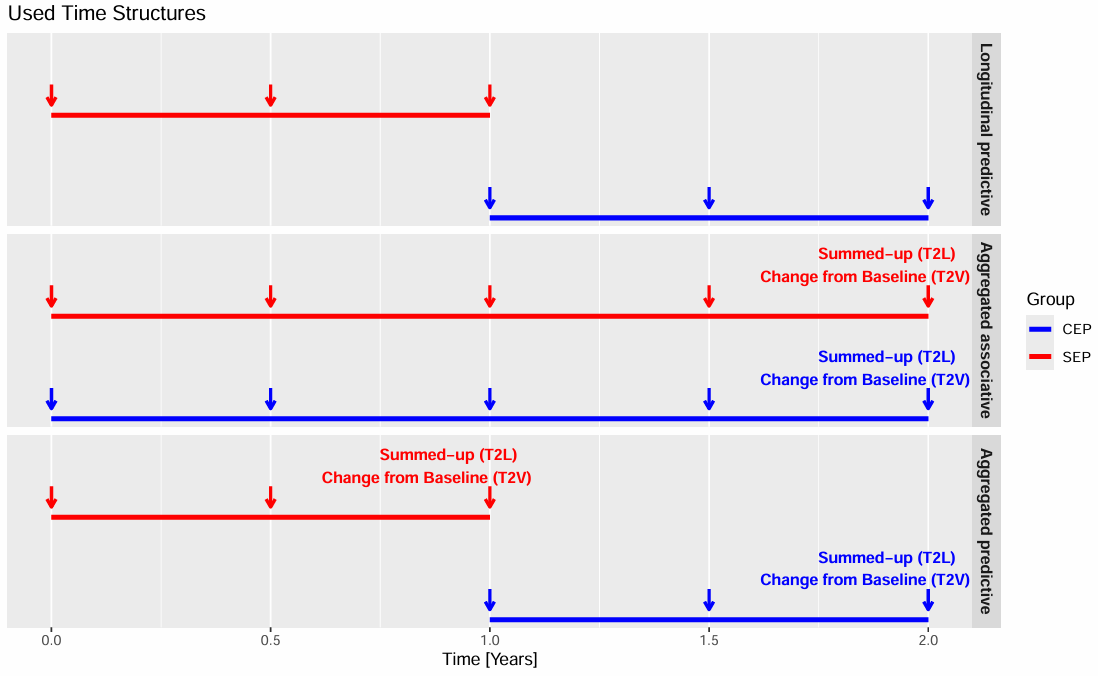
**

**Figure S1:** Different time association settings between SEP and CEP used in the sensitivity analysis

A sensitivity analyses illustrating the individual-level surrogacy (ILS) were conducted by calculating the likelihood reduction factor (LRF) across different temporal association settings between SEP (new/enlarged T2 lesions - T2L - and T2 volume – T2V) and CEP (new relapses): *longitudinal predictive* (repeatedly measured SEP during the first year of the trial and repeatedly measured CEP during the second year) *aggregated associative* (summed-up SEP and CEP), and *aggregated predictive* (summed-up SEP at months 6 and 12, summed-up CEP within the second year). These analyses are restricted to data collected within the first two years of follow-up. Only trials with a duration of at least two years were included.
