# Supplementary material for: Individual-level surrogacy of MRI lesions for disease severity in RRMS: Methods to quantify predictive power and their application to longitudinal data from recent trials
Source: PLoS One. 2025 Dec 26;20(12):e0337893. doi: 10.1371/journal.pone.0337893 (PMC12742783; doi:10.1371/journal.pone.0337893)
Supplement: S2 Fig — Illustration of associative and predictive relationships between a surrogate endpoint (SEP) and a clinical endpoint (CEP) over time considering treatment. In the associative relationship (left), SEP and CEP are associated simultaneously, but values of former measurement time points influence following values (for example, the first value of the SEP influences the second one of the SEP and, when associated, also of the second value of the CEP). In contrast, the prognostic model (right) assumes SEP is measured before the CEP. The associative setting (left) corresponds to the main analysis described in the main article, whereas the predictive setting (right) represents the longitudinal predictive scenario considered in the sensitivity analysis. The trajectories of SEP and CEP are shown for both treatment and control groups over a 2-year period, highlighting differing temporal dynamics in both models. (DOCX) [file pone.0337893.s010.docx]

**
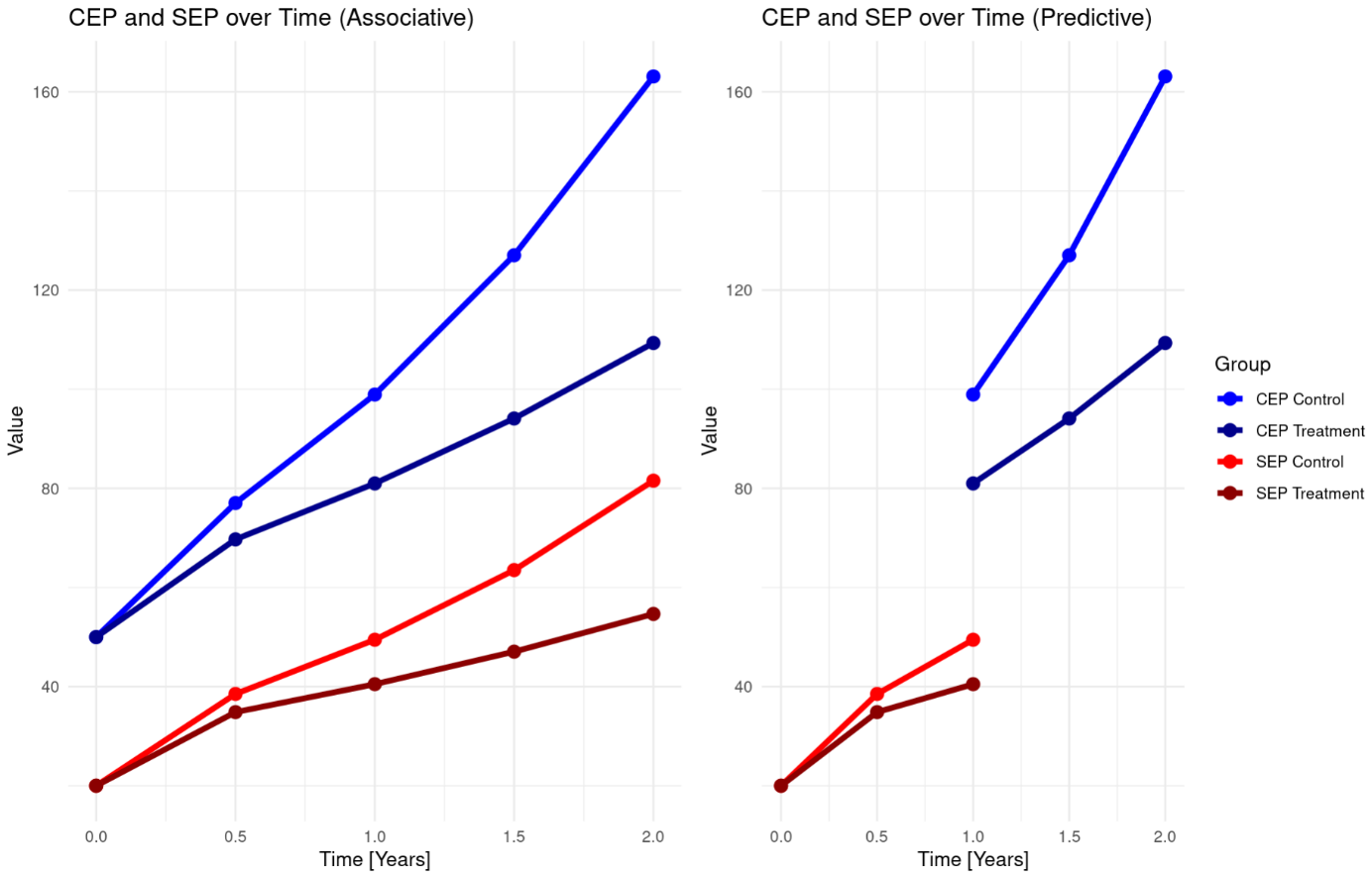
**

**Figure S2:** Different time associations between SEP and SEP

Illustration of associative and predictive relationships between a surrogate endpoint (SEP) and a clinical endpoint (CEP) over time considering treatment. In the associative relationship (left), SEP and CEP are associated simultaneously, but values of former measurement time points influence following values (for example, the first value of the SEP influences the second one of the SEP and, when associated, also of the second value of the CEP). In contrast, the prognostic model (right) assumes SEP is measured before the CEP. The associative setting (left) corresponds to the main analysis described in the main article, whereas the predictive setting (right) represents the *longitudinal predictive* scenario considered in the sensitivity analysis. The trajectories of SEP and CEP are shown for both treatment and control groups over a 2-year period, highlighting differing temporal dynamics in both models.
