# Supplementary figures and images for: Individual-level surrogacy of MRI lesions for disease severity in RRMS: Methods to quantify predictive power and their application to longitudinal data from recent trials
Source: PLoS One. 2025 Dec 26;20(12):e0337893. doi: 10.1371/journal.pone.0337893 (PMC12742783; doi:10.1371/journal.pone.0337893)

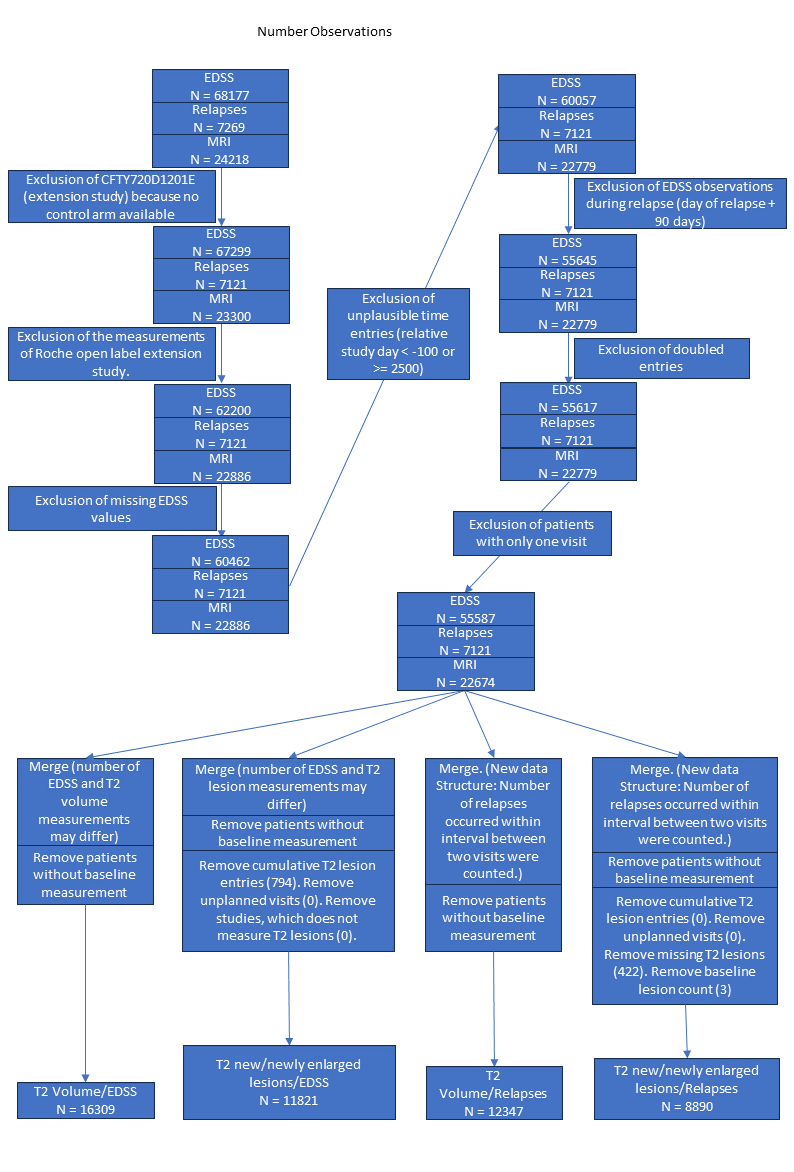


**Figure S*3:*** Flow Chart on number of observations

Supplement: S3 Fig — (DOCX) [file pone.0337893.s011.docx]

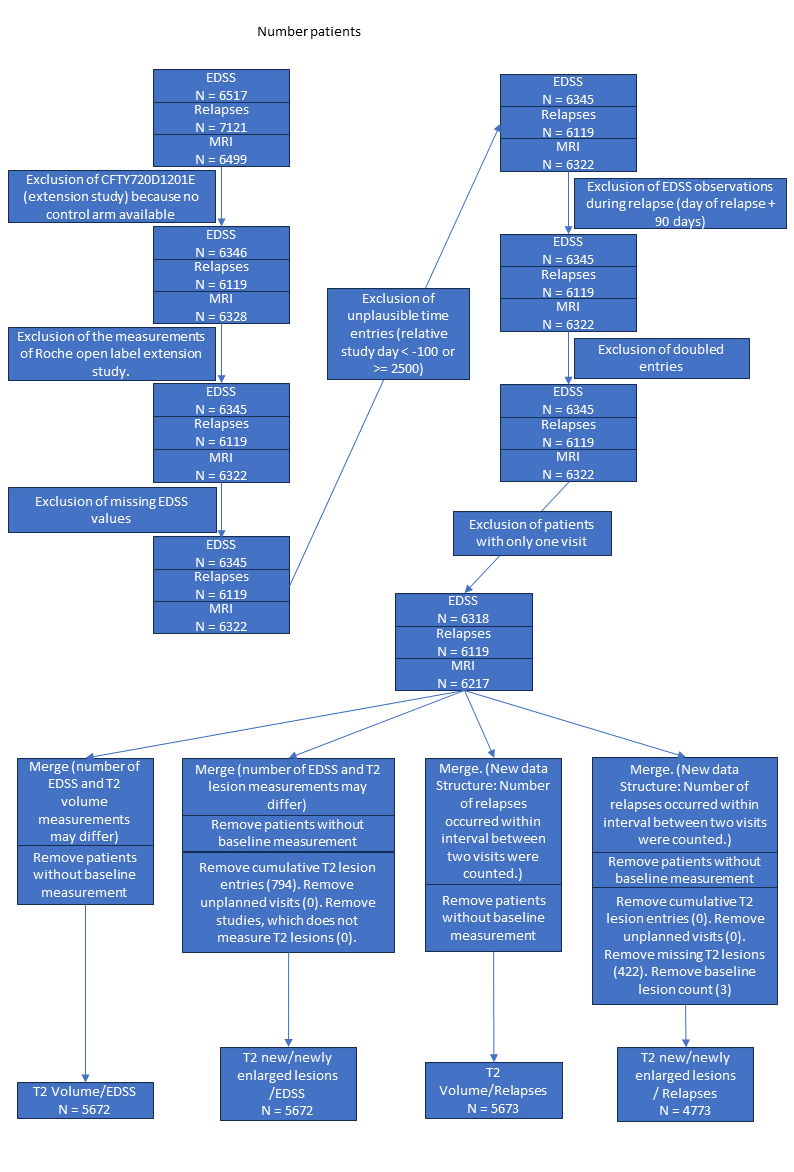


**Figure S4*:*** Flow Chart on number of patients

Supplement: S4 Fig — (DOCX) [file pone.0337893.s012.docx]
