# Supplementary material for: Individual-level surrogacy of MRI lesions for disease severity in RRMS: Methods to quantify predictive power and their application to longitudinal data from recent trials
Source: PLoS One. 2025 Dec 26;20(12):e0337893. doi: 10.1371/journal.pone.0337893 (PMC12742783; doi:10.1371/journal.pone.0337893)
Supplement: S5 Fig — Results from the simulation study (with n = 1000 iterations) utilizing the information-theoretic approach are presented. Red horizontal lines in the figures represent the true LRF values, and the numbers at the top of the panels indicate instances of convergence issues during the simulation study. To derive the LRF, Gaussian, Negative Binomial, Poisson, zero-inflated Poisson, and ordinal models were employed. Datasets with 100, 300, and 600 simulated subjects, each with two or four measurement time points, were generated. Four combinations of considered SEPs and CEPs were considered: 1) Gaussian – Gaussian, 2) Gaussian – Poisson, 3) Poisson – Gaussian, and 4) Poisson – Poisson. Abbreviations: LRF, Likelihood Reduction Factor; Gaus., Gaussian; NB negative Binomial; ZI. Poisson, Zero Inflated Poisson. (DOCX) [file pone.0337893.s013.docx]

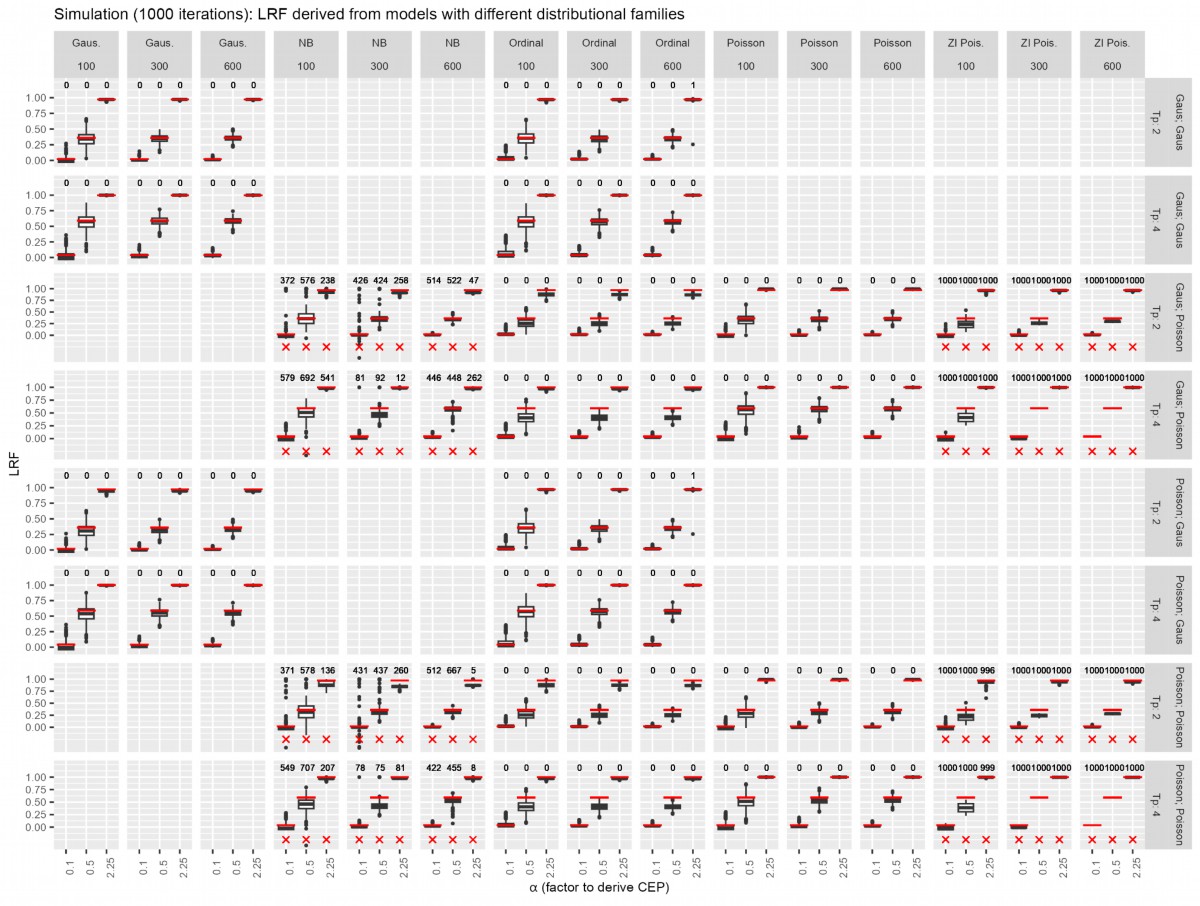


**Figure S5:** Simulation (1000 iterations): Likelihood reduction factor derived from models with different families

Results from the simulation study (with n = 1000 iterations) utilizing the information-theoretic approach are presented. Red horizontal lines in the figures represent the true LRF values, and the numbers at the top of the panels indicate instances of convergence issues during the simulation study. To derive the LRF, Gaussian, Negative Binomial, Poisson, zero-inflated Poisson, and ordinal models were employed. Datasets with 100, 300, and 600 simulated subjects, each with two or four measurement time points, were generated. Four combinations of considered SEPs and CEPs were considered: 1) Gaussian – Gaussian, 2) Gaussian – Poisson, 3) Poisson – Gaussian, and 4) Poisson – Poisson.

Abbreviations: LRF, Likelihood Reduction Factor; Gaus., Gaussian; NB negative Binomial; ZI. Poisson, Zero Inflated Poisson
