# Supplementary material for: Individual-level surrogacy of MRI lesions for disease severity in RRMS: Methods to quantify predictive power and their application to longitudinal data from recent trials
Source: PLoS One. 2025 Dec 26;20(12):e0337893. doi: 10.1371/journal.pone.0337893 (PMC12742783; doi:10.1371/journal.pone.0337893)
Supplement: S6 Fig — Results from the simulation study (with n = 1000 iterations) using the information-theoretic approach are presented. The displayed information represents the absolute difference between the generated and true LRF. To derive the LRF, Gaussian, Negative Binomial, Poisson, zero-inflated Poisson, and ordinal models were employed. Simulated datasets were created with 100, 300, and 600 subjects, each having two or four measurement time points. Four SEP – CEP combinations were considered, symbolized by different colors: 1) Gaussian – Gaussian, 2) Gaussian – Poisson, 3) Poisson – Gaussian, and 4) Poisson – Poisson. Abbreviations: LRF, Likelihood Reduction Factor; Gaus., Gaussian; NB negative Binomial; ZI. Poisson, Zero Inflated Poisson. (DOCX) [file pone.0337893.s014.docx]

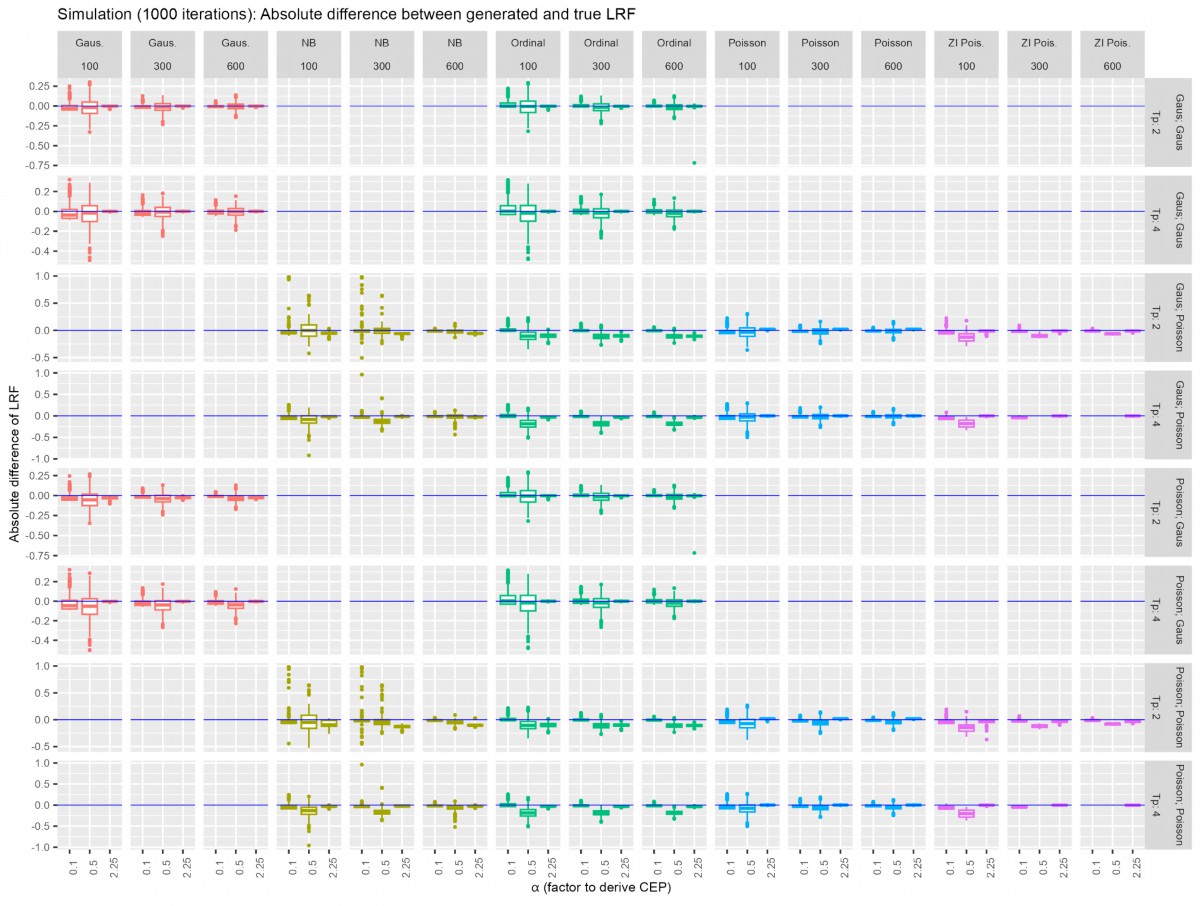


**Figure S6*:*** Simulation (1000 iterations): Absolute difference between generated and true LRF using several model families

Results from the simulation study (with n = 1000 iterations) using the information-theoretic approach are presented. The displayed information represents the absolute difference between the generated and true LRF. To derive the LRF, Gaussian, Negative Binomial, Poisson, zero-inflated Poisson, and ordinal models were employed. Simulated datasets were created with 100, 300, and 600 subjects, each having two or four measurement time points. Four SEP – CEP combinations were considered, symbolized by different colors: 1) Gaussian – Gaussian, 2) Gaussian – Poisson, 3) Poisson – Gaussian, and 4) Poisson – Poisson.

Abbreviations: LRF, Likelihood Reduction Factor; Gaus., Gaussian; NB negative Binomial; ZI. Poisson, Zero Inflated Poisson
