# Supplementary material for: Individual-level surrogacy of MRI lesions for disease severity in RRMS: Methods to quantify predictive power and their application to longitudinal data from recent trials
Source: PLoS One. 2025 Dec 26;20(12):e0337893. doi: 10.1371/journal.pone.0337893 (PMC12742783; doi:10.1371/journal.pone.0337893)
Supplement: S7 Fig — Results from the simulation study (with n = 1000 iterations) using the information-theoretic approach are presented. The displayed information represents the absolute difference between the generated and true LRF. To derive the LRF, Gaussian, Poisson, or ordinal models were employed. Simulated datasets were generated with 100, 300, and 600 subjects, each having two or four measurement time points. It’s important to note that results from the Poisson family were analyzed in their untransformed state. Four SEP – CEP combinations were considered: 1) Gaussian – Gaussian, 2) Gaussian – (transformed) Poisson, 3) transformed Poisson – Gaussian, and 4) transformed Poisson – (transformed) Poisson. Abbreviations: LRF, Likelihood Reduction Factor; tr., transformed; Pois, Poisson. (DOCX) [file pone.0337893.s015.docx]

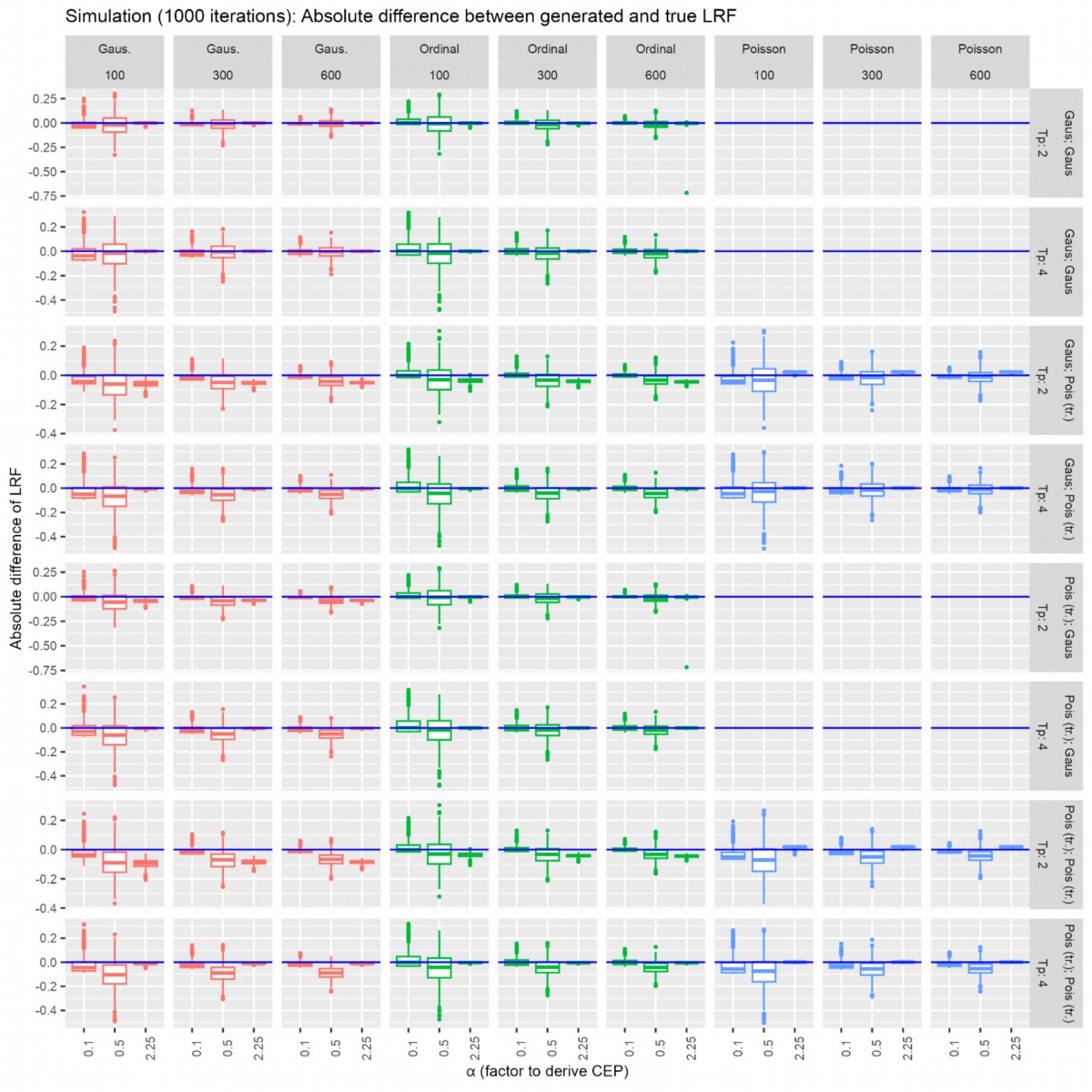


**Figure S7:** Simulation (1000 iterations): Absolute difference between generated and true LRF (transformed outcomes)

Results from the simulation study (with n = 1000 iterations) using the information-theoretic approach are presented. The displayed information represents the absolute difference between the generated and true LRF. To derive the LRF, Gaussian, Poisson, or ordinal models were employed. Simulated datasets were generated with 100, 300, and 600 subjects, each having two or four measurement time points. It's important to note that results from the Poisson family were analyzed in their untransformed state. Four SEP – CEP combinations were considered: 1) Gaussian – Gaussian, 2) Gaussian – (transformed) Poisson, 3) transformed Poisson – Gaussian, and 4) transformed Poisson – (transformed) Poisson.

Abbreviations: LRF, Likelihood Reduction Factor; tr., transformed; Pois, Poisson
