# Supplementary material for: Individual-level surrogacy of MRI lesions for disease severity in RRMS: Methods to quantify predictive power and their application to longitudinal data from recent trials
Source: PLoS One. 2025 Dec 26;20(12):e0337893. doi: 10.1371/journal.pone.0337893 (PMC12742783; doi:10.1371/journal.pone.0337893)
Supplement: S8 Fig — Abbreviations: CEP, Clinical Endpoint. RΛ2 is calculated in different correlation settings when the correlation between SEP and CEP emerges after half of the time points considered (correlation for uneven time points begins at the number of time points/2 - 0.5). Here, α represents the factor used to derive CEP, and ∈ corresponds to the error in CEP, defined as CEP = α* SEP + ∈. (DOCX) [file pone.0337893.s016.docx]

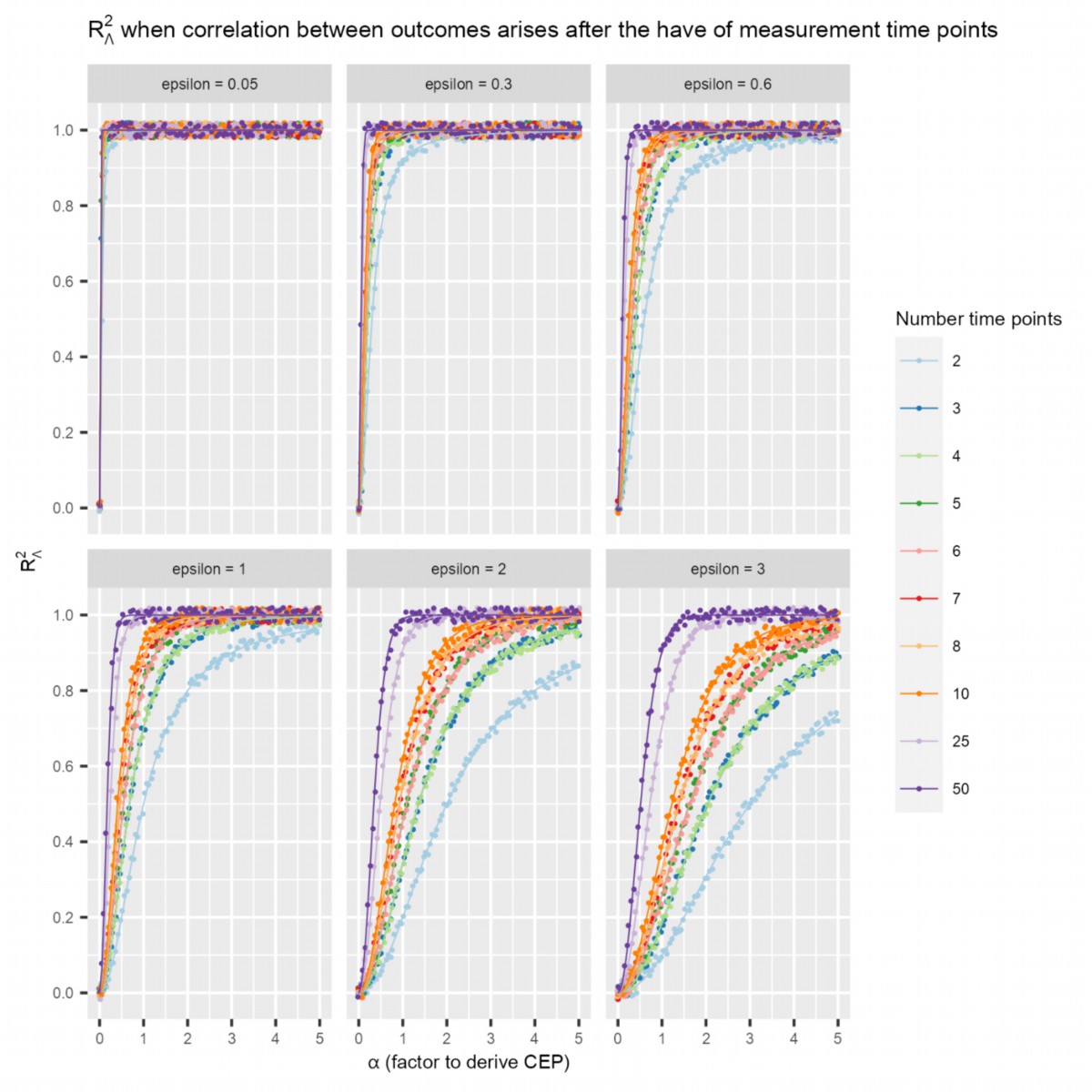


**Figure S8:** $R_{\Lambda}^{2}$ when correlation between outcomes arises after the halve of measurement time points

Abbreviations: CEP, Clinical Endpoint.

$R_{\Lambda}^{2}$ is calculated in different correlation settings when the correlation between SEP and CEP emerges after half of the time points considered (correlation for uneven time points begins at the number of time points/2 - 0.5). Here, $\alpha$ represents the factor used to derive CEP, and $\epsilon$ corresponds to the error in CEP, defined as $CEP = \alpha* SEP + \epsilon$.
