# Supplementary material for: Individual-level surrogacy of MRI lesions for disease severity in RRMS: Methods to quantify predictive power and their application to longitudinal data from recent trials
Source: PLoS One. 2025 Dec 26;20(12):e0337893. doi: 10.1371/journal.pone.0337893 (PMC12742783; doi:10.1371/journal.pone.0337893)
Supplement: S9 Fig — Abbreviations: LRF, Likelihood Reduction Factor; EDSS, expanded disease status scale. LRFs were computed for each trial arm combination (intervention vs. control) using the information-theoretic approach by Alonso and colleagues. To derive the LRF, Gaussian, Negative Binomial, Poisson, zero-inflated Poisson, and ordinal models were utilized. Four SEP – CEP combinations were considered: 1) T2 Volume – EDSS, 2) transformed T2 lesion count – transformed relapses, 3) T2 Volume – transformed relapses, and 4) transformed T2 lesion count – EDSS. Blue or red asterisks indicate convergence problems of one or both regression models from which the LRFs were derived. Notably, the content in column 3 of Figure 2 and column 4 of Fig S9, both utilizing the Poisson distribution family, is identical. Furthermore, the combination of T2V and EDSS outcomes remains consistent between Figure 2 and Fig S9. (DOCX) [file pone.0337893.s017.docx]

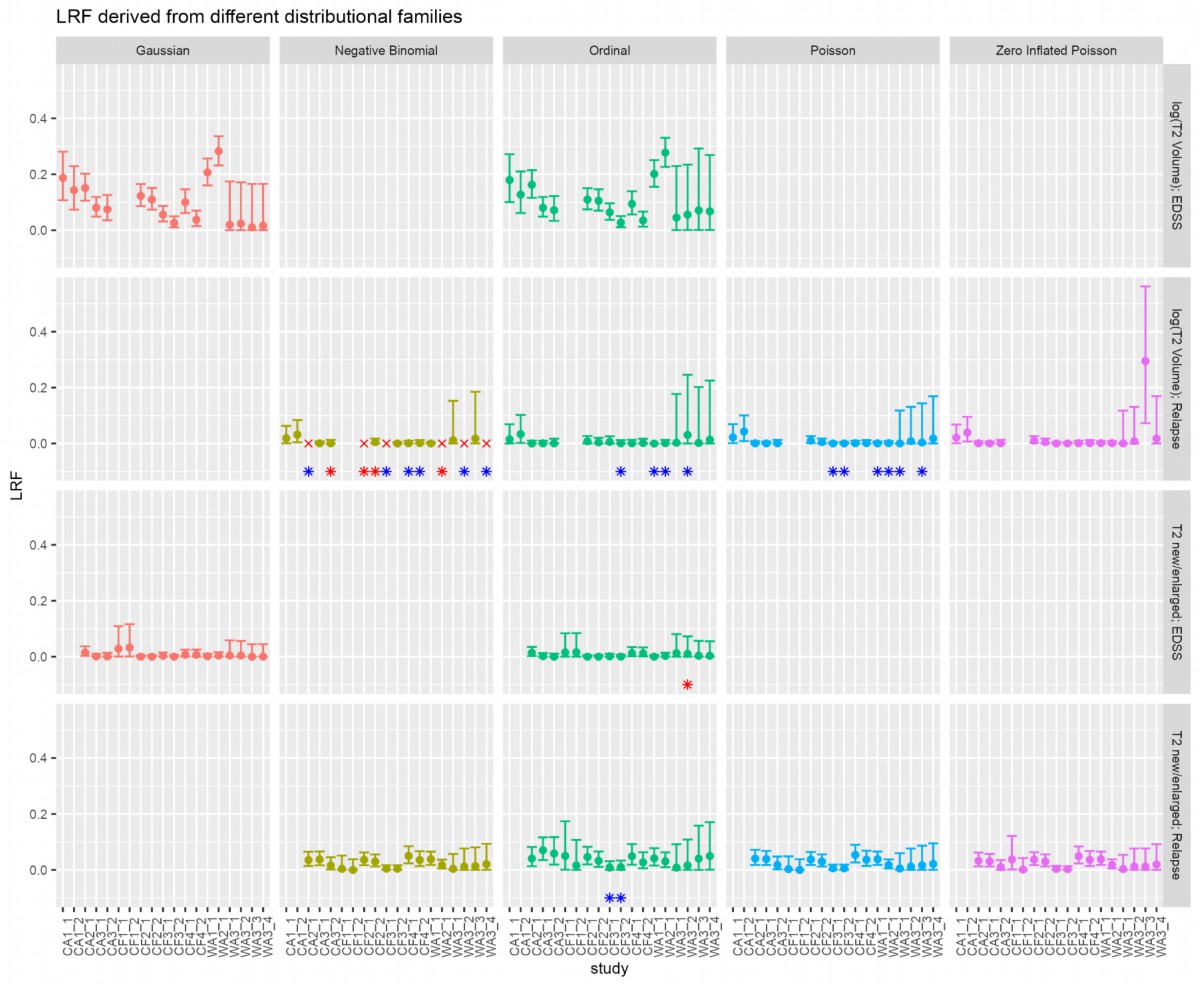


**Figure S9*:*** Likelihood reduction factor derived from different distributional families

Abbreviations: LRF, Likelihood Reduction Factor; EDSS, expanded disease status scale.

LRFs were computed for each trial arm combination (intervention vs. control) using the information-theoretic approach by Alonso and colleagues. To derive the LRF, Gaussian, Negative Binomial, Poisson, zero-inflated Poisson, and ordinal models were utilized. Four SEP – CEP combinations were considered: 1) T2 Volume – EDSS, 2) transformed T2 lesion count – transformed relapses, 3) T2 Volume – transformed relapses, and 4) transformed T2 lesion count – EDSS. Blue or red asterisks indicate convergence problems of one or both regression models from which the LRFs were derived. Notably, the content in column 3 of Figure 2 and column 4 of Fig S9, both utilizing the Poisson distribution family, is identical. Furthermore, the combination of T2V and EDSS outcomes remains consistent between Figure 2 and Fig S9.
