# Supplementary material for: Individual-level surrogacy of MRI lesions for disease severity in RRMS: Methods to quantify predictive power and their application to longitudinal data from recent trials
Source: PLoS One. 2025 Dec 26;20(12):e0337893. doi: 10.1371/journal.pone.0337893 (PMC12742783; doi:10.1371/journal.pone.0337893)
Supplement: S10 Fig — Results of the sensitivity analysis are presented. LRF values derived by the information-theoretic (IT) approach and their 95% confidence intervals (CIs) are displayed in columns one to four, and the proportion treatment effect explained (PTE) values with 95% bootstrap CIs are shown in column five. Blue or red asterisks indicate model convergence issues within the IT methodology, while blue triangles indicate the fulfillment of the Prentice’ criteria. Surrogate outcomes include new/newly enlarged lesions or log (T2 volume) change from baseline and the number of relapses as the clinical endpoint. The aggregated associative approach (rows one and four) evaluates the surrogate and clinical endpoint cross-sectionally after two years. The longitudinal predictive approach (rows two and five) considers surrogate measurements (T2 lesion count or T2 volume change from baseline) at months six and twelve, with the number of relapses within the second year as the clinical endpoint. The aggregated predictive approach (rows three and six) evaluates the surrogate (summed-up T2 lesion count or T2 volume change from baseline) within the first year and the number of relapses within the second year of the trial as the clinical endpoint. The IT approach was utilized to evaluate individual surrogacy. Only trials providing the necessary time structure are included. Abbreviations: LRF, likelihood reduction factor; PTE, proportion explained; ass., aggregated associative setting; long., longitudinal predictive setting; pred., aggregated predictive setting. T2 les., number of new/newly enlarged T2 lesions; T2 Vol., T2 volume; Rel., number of relapses. (DOCX) [file pone.0337893.s018.docx]

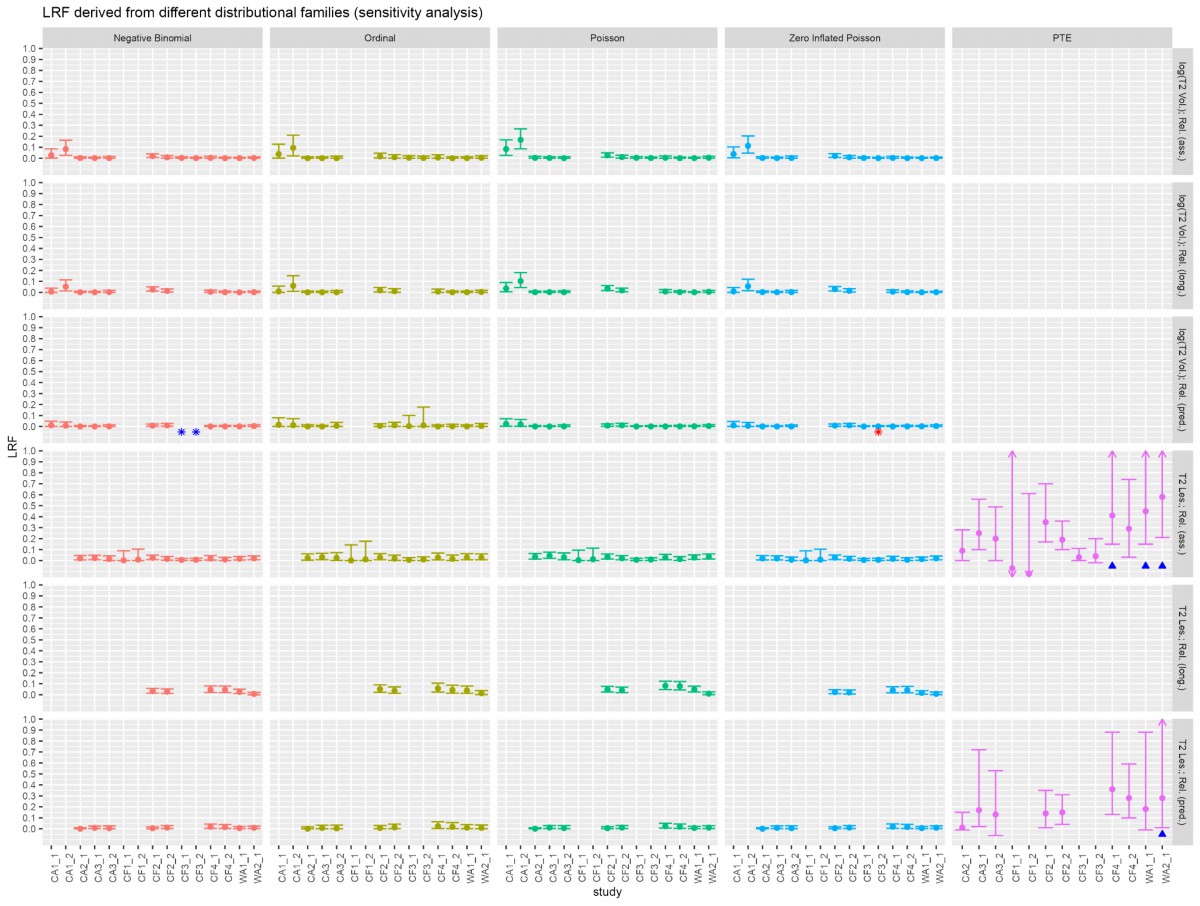


**Figure S10*:*** Sensitivity analysis: Likelihood reduction factor derived from different distributional families

Results of the sensitivity analysis are presented. LRF values derived by the information-theoretic (IT) approach and their 95% confidence intervals (CIs) are displayed in columns one to four, and the proportion treatment effect explained (PTE) values with 95% bootstrap CIs are shown in column five. Blue or red asterisks indicate model convergence issues within the IT methodology, while blue triangles indicate the fulfillment of the Prentice’ criteria. Surrogate outcomes include new/newly enlarged lesions or log (T2 volume) change from baseline and the number of relapses as the clinical endpoint. The *aggregated* *associative* approach (rows one and four) evaluates the surrogate and clinical endpoint cross-sectionally after two years. The *longitudinal predictive* approach (rows two and five) considers surrogate measurements (T2 lesion count or T2 volume change from baseline) at months six and twelve, with the number of relapses within the second year as the clinical endpoint. The *aggregated predictive* approach (rows three and six) evaluates the surrogate (summed-up T2 lesion count or T2 volume change from baseline) within the first year and the number of relapses within the second year of the trial as the clinical endpoint. The IT approach was utilized to evaluate individual surrogacy. Only trials providing the necessary time structure are included.

Abbreviations: LRF, likelihood reduction factor; PTE, proportion explained; ass., *aggregated* *associative* setting; long., *longitudinal predictive* setting; pred., *aggregated* *predictive* setting. T2 les., number of new/newly enlarged T2 lesions; T2 Vol., T2 volume; Rel., number of relapses.
