# Supplementary material for: Individual-level surrogacy of MRI lesions for disease severity in RRMS: Methods to quantify predictive power and their application to longitudinal data from recent trials
Source: PLoS One. 2025 Dec 26;20(12):e0337893. doi: 10.1371/journal.pone.0337893 (PMC12742783; doi:10.1371/journal.pone.0337893)
Supplement: S1 File — (DOCX) [file pone.0337893.s019.docx]

Response to the Reviewers:

Dear Editors and Reviewers,

Thank you very much for the opportunity to revise and resubmit our manuscript. We sincerely appreciate the thoughtful and constructive comments provided by both the reviewers and the editorial team, which have been essential in improving the quality of our work.

In answer to the reviewers' feedback, we prepared a detailed, point-by-point response that outlines how each comment has been addressed. We also ensured that all journal guidelines and formatting requirements have been fully implemented in the revised manuscript.

Thank you again for your time and consideration. We look forward to your response and the opportunity to contribute to your journal.

Sincerely,
the authors

| **Comments of Reviewer 1** | **Answers/Changes in the manuscript** |
| --- | --- |
| Statistical Methodology Concerns The authors' central argument rests on the superiority of the Likelihood Reduction Factor (LRF) over traditional surrogacy measures, but this premise has limitations. The LRF threshold of 0.5 borrowed from oncology may not be appropriate for MS, where the disease mechanisms and treatment effects differ substantially. The authors provide limited justification for why their chosen statistical measure should be considered the gold standard for clinical decision-making. | **Statistical Methodology Concerns:**  We appreciate the reviewer’s thoughtful comments on statistical methodology. In the revised manuscript, we expanded and clarified our rationale for using the Likelihood Reduction Factor (LRF) rather than frequently used surrogacy measures like OR, HR, sensitivity, specificity, or the ill-defined proportion of treatment effect explained (PTE). See text in the background for further clarification.  LRF is not an arbitrary chosen measure for the quality of prediction. It is the generalization of the well-founded R² measure (coefficient of determination) known to describe the extent of prediction error in linear regression. R² close to one is an excellent setting for prediction (nearly no prediction error) while R² of 0 is a fully random prediction setting. The LRF is already known as methodological concept around 2005 but never made it into the medical prediction literature. Our paper tries to provide this bridge and proposes to use LRF as a quality measure for surrogacy issues in medicine.  Introducing a threshold is a natural thing to do. We provide an example from the setting of added value regulation. The IQWIG defines R²=0.5 as the lower limit for useful surrogates in oncology. We support the statement of the reviewer that the MS community needs to find thresholds which serve their needs. Our future research will try to tackle the threshold problem by looking at concepts proposed by Wim van der Elst:  Van der Elst, Wim, et al. "The individual‐level surrogate threshold effect in a causal‐inference setting with normally distributed endpoints." Pharmaceutical Statistics 20.6 (2021): 1216-1231.  **Why LRF is a useful metric:**  We clarified in Line 114–117 that “The threshold defines a minimal quality measure above which the metric is considered clinically useful. For example, an SEP with a corresponding LRF value above a pre-defined threshold may be indicative for treatment failure” This shows that our use of LRF is intended to support a qualitative judgement on whether a factor is informative or not.  Our intention is to present the LRF as a relevant tool that contributes to the utility of SEPs in practice. By quantifying the reduction of uncertainty in individual predictions, the LRF provides clinicians with a clearer indication of whether a SEP meaningfully improves prognostic accuracy. This focuses on variability and clinical interpretability complements, rather than replaces existing clinical assessments in RRMS. We therefore added the following explanation in lines 204 - 209: “From a clinical perspective, the LRF expresses how much percentage of the uncertainty in an individual prediction is explained by considering the SEP. A higher reduction means that the prediction becomes more precise, for example when the expected range of a deterioration in EDSS values is narrower instead of wide. As mentioned above, a low variability in individual predictions is essential for informed clinical decision making.”  We further addressed the risk of misleading metrics by explicitly adding lines 96–100 “However, a large OR does not help to quantify individual risk precisely, as it reflects a relative ratio which transforms a baseline risk. Different individual baseline risks give different individually transformed risks even for the same OR. If we have a risk of 10% or 30% for a severe disease course in the group of a favorable SEP, the respective risks are 18% and 46% in the group of unfavorable SEP given an OR of 2.” In the discussion we added the following sentence (lines 492 - 496) “We showed that in the MS literature on individual prediction, mainly population-wide association measures were used. In most cases significant PTEs, ORs, and HRs (see Table S1) were reported instead of metrics suited for assessing the quality of predictions at the individual level, such as PPVs, NPVs, calibration, correlation, or the LRF.”. Furthermore, we reformulated a paragraph in the discussion (lines 472–489) to make the provided examples easier to interpret and assess.  Finally, we provide a schematic assessment of the metrics used in two relevant MS guideline articles on the clinical usability of existing treatment response tools. To emphasize this, we added a sentence in lines 218 - 220: “5) As a practical application we provide an assessment of the evidence of T2L as an individual-level SEP and treatment failure indicator based on literature cited in two MS guideline articles.”  **On the use of thresholds:**  In the revision we also emphasise (see lines 117–121) that, although correlation coefficients (r) and R² values are reported in the MS literature, this concept of applying pre-defined thresholds for clinical utility is also rarely used in MS literature. To our knowledge, no studies have validated appropriate thresholds for r or R² in MS. As stated in Line 120–121, “To define such a threshold for MS is out of the scope of this article.” We therefore do not propose a new threshold for R² or LRF values in MS but rather demonstrate that the LRF is a candidate measure because it represents a generalisation of R², which is (along with the r) used in MS literature. To help clinicians gain an intuitive understanding of this metric, we added the following sentence to the discussion (lines 449 - 457): “LRFs were mostly below 0.2 indicating that in most examined scenarios less than 20% of the CEP variability is explained by the SEP. This means that the SEP provides relatively little information for predicting the CEP on the individual level, and such a level of explained variability may be insufficient to support informed treatment decisions. To provide an intuitive understanding of the LRF, Figs A and B in S1 text may serve as illustrative examples. While data variability is low in Fig A and high in Fig B, this difference is reflected in the R² and LRF. Overall, the scenario shown in Fig A provides a more suitable basis for individual predictions than that in Fig B.”  **On external validation**  To further emphasize the importance of external validation of prediction algorithms, we added in the revised manuscript the following sentence (lines 154 - 157): “External validation is the state-of-the-art, but rarely provided for prediction models in the RRMS literature^7,8^.This means that the generalisability of the evaluated prediction models to new persons remains unproven, and their clinical applicability cannot be assumed” Our revision now makes clear that prediction models for RRMS are rarely externally validated, which limits their generalisability and clinical applicability. One objective of our study was to examine clinical trial data to determine whether the optimistic predictive claims frequently reported in the literature can be substantiated. See the following two guidelines on the development of validated prediction models:  Collins, Gary S., et al. "Evaluation of clinical prediction models (part 1): from development to external validation." Bmj 384 (2024).  Riley, Richard D., et al. "Evaluation of clinical prediction models (part 2): how to undertake an external validation study." Bmj 384 (2024).  By incorporating these clarifications, the revised manuscript (a) explains why the LRF is clinically informative, (b) clearly states that defining an MS-specific threshold is beyond the scope of our article, and (c) emphasises the importance of external validation. |
| The simulation studies reveal concerning biases, particularly underestimation with smaller samples and numerical instability in multiple scenarios. Given that real-world clinical populations often have characteristics similar to the problematic simulation conditions, this raises questions about the reliability of the method in practice. | We thank the reviewer for pointing out the potential biases observed in our simulation studies. We therefore emphasized more clearly in the manuscript that, in our assessment, there is no systematic bias present in the simulation. We show bias in specific settings.  **On the bias**  Figure S7 corresponds to the results shown in Figure 2. When both SEP and CEP are normally distributed (Gaussian-Gaussian combinations; Figure S7 columns 1-3, rows 1-2), the bias is minimal, as the median of the simulated LRFs closely matches the true value. Variability is higher in scenarios with smaller sample sizes or moderate associations between SEP and CEP.  When either the SEP or CEP endpoint was transformed (e.g., for count variables such as T2L or relapse counts), bias increased, and the true LRF values were underestimated by approximately 0.1 (see the median LRF values in columns 1–3, rows 4–8). Similar patterns were observed for ordinal endpoints (columns 4–6). Using a Poisson model for non-transformed count endpoints resulted in underestimation only when both SEP and CEP were counts (columns 7–9, rows 7–8).  Applying these observations to Figure 2: for T2 lesion volume and EDSS, both endpoints are approximately Gaussian (column 1, row 3), corresponding to the scenario with the lowest bias in our simulations. Even in this case, LRFs were mostly below 0.15 and always below 0.3. Furthermore, most of the 10 included studies have sample sizes above 300, with six studies above 600 (range 333–1638; see Table 1), corresponding to medium and large sample size scenarios in the simulation. Even studies with N = 1638 show LRFs below 0.1 (see Figure 2, CF3_1 and CF3_2). LRF values remain low even in scenarios with bias and potential underestimation of up to 0.104 having minimal impact on the overall interpretation.  Overall, these results suggest that while small sample sizes or non-normal endpoints can lead to bias, the method remains informative, and in practical settings with moderate to large sample sizes and approximately normal endpoints, the LRF provides a reliable measure for assessing the predictive value of SEPs.  To clarify these points, we added the following text in lines 383-385 (Results), 458–464 (Discussion), and line 567 - 572 (Conclusion):  “When both SEP and CEP are normally distributed, the LRF shows minimal bias, though smaller sample sizes or transformed endpoints can underestimate its true value (Fig S7).  “As shown in Fig S7, this limited predictive power should also be interpreted in light of potential bias. While scenarios with transformed or non-normal endpoints tend to underestimate the true LRF (up to -0.104 for results corresponding to Fig 2), the bias is minimal when both SEP and CEP are approximately normal, and sample sizes are moderate to large (N ≥300). Accordingly, the LRF values for T2V and EDSS (Fig 2, row 3) are not biased, while values in other scenarios may be underestimated. Even so, LRFs remain low.”  “To quantify ILS, we introduced the LRF as a reliable and generalized ILS quality measure for normally distributed longitudinal data. In some scenarios where endpoints followed a Poisson distribution, were ordinal, or had been transformed, the LRFs were underestimated by up to 0.104. However, this did not affect the overall interpretation of the LRF values derived from the ten clinical trials.”  **On numerical instability**  The numerical issues shown in Figure S5 (NB and ZI Poisson) arise from the simulation design, where data were first generated as Gaussian and then transformed into Poisson-distributed variables. As expected, negative binomial (NB - columns 4-6) or zero inflated (ZI Pois. – columns 13-15) analysis methods do not fit to the simulated data.  The instabilities observed in Figure 2, column 3 (results using Poisson models to the data) suggest that the distribution of the number of relapses given T2V (P [Number relapses \| T2L]) does not always follow a Poisson distribution. Ordinal models, in general, are not prone to numerical instability. Nevertheless, numerical instabilities have minimal impact, as the LRF values across the model families used to derive them are similar (compare columns 1 with 2 and 3 in Fig 2).  We added the following text to the results part of the revised manuscript (lines 402 - 409): “The instabilities observed in Fig 2 (blue asterisks) indicate that the distribution of the number of relapses given T2V (P [Number relapses \| T2L]) does not always perfectly follow a Poisson distribution. Since ordinal models are generally more prone to numerical instability, our results suggest that models with Gaussian-Gaussian endpoint combinations appear more reliable. Nevertheless, the observed numerical instabilities had minimal impact, as LRF values were highly consistent across model families with and without instability, showing comparable results between Gaussian, Poisson, and ordinal models (compare columns 1 to 3 in Fig 2).”; to the discussion we added following sentence (lines 464 - 466): “Overall, the results suggest that Gaussian-Gaussian endpoint combinations provide unbiased and less numerically instable estimates compared to Poisson or ordinal models.”  In general, we employ well established methodologies for surrogate endpoint evaluation (introduced before 2005) and conducted simulation studies (Figure S7) to assess potential bias under different endpoint distributions and sample sizes, a step not previously done for other commonly used metrics (e.g. PTE). |
| Clinical Relevance Issues The most significant weakness is the disconnect between statistical surrogacy measures and clinical utility. The authors focus heavily on population-wide measures but provide insufficient discussion of how LRF values translate to individual patient care. A statistical measure showing weak surrogacy doesn't necessarily invalidate clinical utility if the surrogate still provides actionable information for treatment decisions. | We agree with the reviewer on the importance of providing statistical measures which are related to clinical reality. However, as discussed in the background section of our paper, the MS literature is full of such disconnects. No paper mentions the clinical meaning of a significant correlation between an SEP and a CEP and no paper explains the clinical meaning of a significant OR or HR. For us the clinical connection is represented if the prediction is precise. A precise prediction of an unfavorable clinical event is actionable. One should ensure the event does not happen. Furthermore, our study does not invalidate the clinical use of MRI or T2 lesions for disease monitoring but suggests that over periods of up to five years, T2 lesions provide limited predictive value for individual disease progression or treatment failure in RRMS. Population-level trends may remain informative, yet reliable individual predictions require evaluation using PPV, NPV, calibration, AUC, and internal and external validation. While we do not propose alternative monitoring tools, our work highlights key methodological considerations for identifying robust surrogate endpoints. To emphasize this, we added the following two sentences to the implications (lines 577 - 579 and 592 - 593): “However, we would like to clarify that our study does not question the clinical use of MRI or T2L monitoring but indicates that T2L provides limited predictive value for individual RRMS outcomes.”; “Research may focus on robust individual-level evaluation using appropriate metrics and methods to identify reliable SEPs for RRMS outcomes.”  A high LRF tells us that an individual prediction is mostly precise, a low LRF tells us the contrary. The individual may not have an advantage from clinical action if the precision of the prediction is low. Conceptually, the LRF allows estimation of how informative a surrogate is for individual-level prediction. This supports clinical decision-making aimed at maximizing treatment precision.  To clarify this point, we expanded the manuscript to illustrate how LRF can be used as a clinically meaningful metric (lines 526 - 536): “From a clinical perspective, the utility of the LRF quantifying individual-level SEP quality can be illustrated in the following example: Consider a screening test with low sensitivity but high specificity. In this case, positive results are generally reliable (high specificity -> low false positives), but many true cases remain undetected, meaning the test alone is insufficient to guide decisions for all individuals. In a screening context, false positives can be clarified through subsequent testing before any intervention. In contrast, when a therapeutic decision is made directly based on this initial test, there is no opportunity to correct for misclassifications, which underscores the need for robust and reliable individual-level SEPs to guide treatment decisions. However, current evidence on T2L as a SEP detecting for treatment failure is frequently assessed using metrics that are not appropriate for prediction quality.”  Claiming a high ILS quality for the MS related SEPs is like claiming that there are only few false positives or false negatives. In the context of MS, the community often reports associative measures such as odds ratios, hazard ratios, or the proportion of treatment effect explained, whereas measures suited for individual-level prediction - such as PPVs, NPVs, calibration, AUC, or the LRF - are not standard. |
| The use of EDSS changes rather than confirmed disability progression is problematic, as acknowledged by the authors. EDSS changes are notoriously variable and may not capture the clinically meaningful disability outcomes that matter for long-term prognosis. | We thank the reviewer for this important comment. Our choice of EDSS and the annualized relapse rate as endpoints was driven by the data available in the clinical trials included in our study. The surrogate role of T2 lesions, which our work assesses, is also based on these commonly reported endpoints. Our intention was not to propose or validate new surrogate markers, but rather to critically evaluate the predictive value of established measures. We acknowledge that more recent or composite endpoints (e.g., confirmed disability progression, brain atrophy, cognitive measures) may better capture the pathogenesis of RRMS, and their investigation represents an important avenue for future research.  Similarly, long-term prognosis could not be analyzed due to the limitations of the trial data, but we plan to explore this in future studies using registry datasets that provide extended follow-up. |
| Study Design Limitations  The "short observation periods" mentioned by the authors are particularly concerning for a disease like MS where meaningful clinical outcomes may take years to manifest.  The heterogeneity across trials (different imaging standards, observation periods, populations) introduces substantial noise that may obscure genuine surrogacy relationships.  The exclusion of patients with missing data rather than using appropriate imputation methods could introduce systematic bias, particularly if missingness relates to disease activity or outcomes. | We thank the reviewer for highlighting these important concerns.  **Observational period:**  It is true that meaningful clinical outcomes in MS often take years to manifest. However, long-term (observational) studies are generally scarce in the MS community (see Table S1), and clinical trials frequently assume shorter observation periods. In our study, the maximum observation period was five years, which is at the upper range compared to most MS trials and therefore provides relevant information, particularly for people who develop lesions early. In addition, shorter observation periods as used by us are also used in studies which are mentioned in papers behind the MAGNIMS and CMSWG guidelines (see Table S1). While observational, registry, or routinely collected data studies may provide longer follow-up, these typically come at the cost of lower data quality including different biases and heterogeneity.  **Heterogeneity:**  LRFs were calculated separately for each trial. In general, clinical trials adhere to strict protocols and standardized reading guidelines for MRI assessments, ensuring consistency and reliability of imaging data across study sites.  To clarify this point, we added following sentence to the limitations (lines 555 - 557): “T2 imaging standards across the trials were not established. However, internally, there were reading guidelines for MRI images. Accordingly, potential heterogeneity in T2 lesion detection and assessment between trials may be present.”  **Exclusion of participants:**  Regarding missing data, only individuals were excluded if they had no longitudinal data (i.e., only a single measurement) or if the baseline measurement was missing. The exclusion of missing EDSS or MRI values did not affect the overall patient count (see patient-level flowchart Figure S4), allowing us to assume that the data were missing at random, which would not introduce bias. Excluding individuals with only one visit affected 27 patients for EDSS and 105 for MRI assessments, corresponding to less than 1.7% (in the case of MRI: 105/6322). Most exclusions occurred when an outcome measurement (e.g., EDSS) could not be paired with a corresponding surrogate measurement (e.g., new T2 lesions). Potential informative missing may be present (missing related to the outcomes), but this cannot be addressed by proposed imputation methods. |
| Interpretation Problems The authors' conclusion that "existing surrogacy claims should be reassessed" is overly strong given their methodological limitations. Their negative findings could reflect:  Inappropriate statistical measures for the clinical context Insufficient observation periods for meaningful outcomes Heterogeneity masking real relationships The inherent complexity of MS pathophysiology  Missing Clinical Context The manuscript lacks adequate discussion of how their findings should impact clinical practice. If MRI lesions have limited individual-level surrogacy by their measures, what alternative approaches do they recommend for treatment monitoring? The clinical community's widespread adoption of MRI monitoring reflects accumulated clinical experience that may not be captured by their statistical framework. | Thank you for this crucial information. We agree with the reviewer here that our work can only be a first step towards better understanding individual prediction in MS. The complexity of pathophysiology is indeed an important point and may inspire the development of new surrogate and clinical endpoints. However, predictive models based on such endpoints should adhere to rigorous methodological standards. We believe that the LRF represents a key component within this predictive framework (see below). We added this sentence to the limitations to emphasize the complex pathophysiology of RRMS (lines 549 - 553): “Our analysis focused on EDSS and relapse rate and did not consider composite or more modern endpoints (e.g., PIRA, cognitive measures, patient-reported outcomes), which may result in underestimating the true predictive value of MRI lesions and does not fully capture the pathophysiological complexity of RRMS.”. The inherent complexity of MS is an important point, but it also existed for the research before and did not devalue findings which entered the guidelines. We clarified in the manuscript the points previously mentioned. In particular, the rationale for using the LRF as a measure for prediction precision is presented in more detail, including its methodological advantages over previously used measures in the MS literature. The limitations of the prediction time frames have been addressed in the limitation section. Additionally, the manuscript now more explicitly addresses heterogeneity across clinical trials: each trial follows specific MRI reading guidelines. The context and relevance of these considerations are now more clearly outlined in the revised text. |

| **Comments of Reviewer 2** | **Answers/Changes in the manuscript** |
| --- | --- |
| While the authors demonstrate that LRF is a reliable measure of ILS, the manuscript does not define a clinically meaningful threshold for interpreting LRF values in MS. Without a clear benchmark, it is difficult for readers to judge the clinical implications of “weak” vs “moderate” ILS signals. The discussion references oncology standards (IQWiG cutoff of 0.5), but a more disease-specific rationale would strengthen the paper. | We thank the reviewer for the valuable comment.  In the revision we emphasise (see lines 117–121) that, although correlation coefficients (r) and R² values are reported in the MS literature, this concept of applying pre-defined thresholds for clinical utility is also rarely used in MS literature. To our knowledge, no studies have validated appropriate thresholds for r or R² in MS. As stated in Line 120–121, “To define such a threshold for MS is out of the scope of this article.” We therefore do not propose a new threshold for R² or LRF values in MS but rather demonstrate that the LRF is a candidate measure because it represents a generalisation of R², which is (along with the r) used in MS literature. To help clinicians gain an intuitive understanding of this metric, we added the following sentence to the discussion (lines 449 - 457): “LRFs were mostly below 0.2 indicating that in most examined scenarios less than 20% of the CEP variability is explained by the SEP. This means that the SEP provides relatively little information for predicting the CEP on the individual level, and such a level of explained variability may be insufficient to support informed treatment decisions. To provide an intuitive understanding of the LRF, Figs A and B in S1 text may serve as illustrative examples. While data variability is low in Fig A and high in Fig B, this difference is reflected in the R² and LRF. Overall, the scenario shown in Fig A provides a more suitable basis for individual predictions than that in Fig B.” |
| The analysis relies primarily on EDSS and relapse rate. Both measures have known limitations (EDSS insensitivity to cognitive and subtle functional changes, relapse heterogeneity). The absence of composite outcomes or more modern endpoints (e.g., brain atrophy, patient-reported outcomes, cognitive measures) may underestimate the true predictive value of MRI lesions. This limitation should be more explicitly acknowledged. | We sincerely thank the reviewer for this insightful feedback.  We added this sentence to the limitations to emphasize the complex pathophysiology of RRMS (lines 549 - 553): “Our analysis focused on EDSS and relapse rate and did not consider composite or more modern endpoints (e.g., PIRA, cognitive measures, patient-reported outcomes), which may result in underestimating the true predictive value of MRI lesions and does not fully capture the pathophysiological complexity of RRMS.”  EDSS and relapse rate were common endpoints in the ten clinical trials included in our analysis. These were the clinically relevant outcomes at the time the trials were conducted, and our choice of endpoints was therefore determined by the available data. Alternative or more modern endpoints were unfortunately not accessible within these datasets. |
| The trials included did not follow standardized MRI protocols, which introduces variability in lesion detection. The paper notes this but does not quantify or adjust for it. Exploring how heterogeneity in MRI acquisition influences LRF estimates would add value. | We thank the reviewer for this comment. LRFs were calculated separately for each trial. In general, clinical trials adhere to strict protocols and standardized reading guidelines for MRI assessments, ensuring consistency and reliability of imaging data across study sites.  To clarify this point, we added following sentence to the limitations (lines 555 - 557): “T2 imaging standards across the trials were not established. However, internally, there were reading guidelines for MRI images. Accordingly, potential heterogeneity in T2 lesion detection and assessment between trials may be present.” |
| The rationale for excluding open-label extension data should be expanded. | We thank the reviewer for this comment. We excluded open-label extension data because blinding was lifted in these phases, which could have influenced the assessment of T2 lesions and EDSS by both participants and investigators, potentially introducing bias. We added the following to the methods (lines 287 - 291): “Observations collected during the open label extension of trials NCT01247324, NCT01412333, and NCT00676715 were excluded, as the removal of blinding in these phases could have influenced both patient-reported and investigator-assessed outcomes, including T2 lesion counts and EDSS measurements, potentially introducing bias.” |
| Figures (e.g., simulation results and LRF estimates) are dense and may benefit from simplified summary panels or thresholds to guide interpretation. | Thanks for this suggestion. Unfortunately, this data can no longer be modified, as access to the dataset has expired. |
| The paper could highlight more explicitly how these findings should influence current clinical guideline recommendations (MAGNIMS, CMSWG). | Thank you for this valuable comment.  We addressed this by adding the following sentence (lines 586 - 592):” “To support the validity of guidelines, we recommend to report prospective risk estimates, validate models with calibration and discrimination metrics, define thresholds for predictive usefulness, and assess clinical utility via decision curve analysis in the future^47^. A consensus on such standards paralleling TRIPOD^48^ would improve interpretability, timeliness, and relevance of MRI-based predictions and strengthen guideline recommendations.” |
| “arouse” (p.17, line 373) → should be “arose.” | Thank you for the suggestion. It has been incorporated into the revised manuscript. |
| “proof that the LRF has properties” (p.8, line 176) → should be “proved” or “demonstrated.” | Thank you for the suggestion. It has been incorporated into the revised manuscript. |
| “PwRRMS” is introduced without definition in some places—ensure it is consistently defined as people with RRMS. | Thank you for the suggestion. It has been incorporated into the revised manuscript (PwRRMS = persons with RRMS). |
| Some long sentences in the Background (pp.4–7) could be divided for clarity. For example: | Thank you for the suggestion. It has been incorporated into the revised manuscript. |
| References should be checked for consistent formatting (e.g., italics for journal names, spacing). | Thank you for the suggestion. It has been incorporated into the revised manuscript. |

| **Comments of Editors** | **Answers/Changes in the manuscript** |
| --- | --- |
| Please ensure that your manuscript meets PLOS ONE's style requirements, including those for file naming. The PLOS ONE style templates can be found at  https://journals.plos.org/plosone/s/file?id=wjVg/PLOSOne_formatting_sample_main_body.pdf and [https://journals.plos.org/plosone/s/file?id=ba62/PLOSOne_formatting_sample_title_authors_affiliations.pdf](https://track.editorialmanager.com/CL0/https:%2F%2Fjournals.plos.org%2Fplosone%2Fs%2Ffile%3Fid=ba62%2FPLOSOne_formatting_sample_title_authors_affiliations.pdf/1/010f0199559d055a-51225b04-92db-46b0-a283-de095b17c436-000000/Wuxw6ONxLc33WSIvxmGmjq8uRoX1MDZfAGH2H_qS1Yw=228) | Thank you for the suggestion. It has been incorporated into the revised manuscript. |
| 2. Thank you for stating the following in your manuscript:  [Funding of Begüm Irmak Ön and Stefan Buchka via DIFUTURE (grant number: BMBF 01ZZ1804C) and Joachim Havla by DIFUTURE (grant number: BMBF 01ZZ1804B).]  We note that you have provided funding information that is currently declared in your Funding Statement. However, funding information should not appear in the Acknowledgments section or other areas of your manuscript. We will only publish funding information present in the Funding Statement section of the online submission form.  Please remove any funding-related text from the manuscript and let us know how you would like to update your Funding Statement. Currently, your Funding Statement reads as follows:  [Funding of Begüm Irmak Ön and Stefan Buchka via DIFUTURE (grant number: BMBF 01ZZ1804C) and Joachim Havla by DIFUTURE (grant number: BMBF 01ZZ1804B). The funders did not play a role in the study design, data collection and analysis, decision to publish, or preparation of the manuscript]  Please include your amended statements within your cover letter; we will change the online submission form on your behalf. | Thank you for the suggestion. It has been removed from the revised manuscript. The information on funding is incorporated in the cover letter: “Funding of Begüm Irmak Ön and Stefan Buchka via DIFUTURE (grant number: BMBF 01ZZ1804C) and Joachim Havla by DIFUTURE (grant number: BMBF 01ZZ1804B).” |
| 3. Thank you for stating the following in the Competing Interests section:  [JH : reports a grant for OCT research from the Friedrich-Baur-Stiftung, Amgen/Horizon, Sanofi, Roche, and Merck, personal fees and nonfinancial support from Merck, Alexion, Novartis, Roche, Celgene, Biogen, Bayer, Neuraxpharm and Horizon/Amgen, non-financial support from the Sumaira-Foundation and Guthy-Jackson Charitable Foundation, all outside the submitted work.  BIO: Since February 2025, BIO is employed in Staburo GmbH, a data science company with clients in the biopharma industry. The projects and clients that BIO is involved in are all outside of the scope of the submitted work.].  We note that you may have received funding from these commercial sources: Sanofi, Roche, Merck, Alexion, Novartis, Celgene, Biogen, Bayer, Neuraxpharm  Please provide an amended Competing Interests Statement that explicitly states this commercial funder, along with any other relevant declarations relating to employment, consultancy, patents, products in development, marketed products, etc.  Within this Competing Interests Statement, please confirm that this does not alter your adherence to all PLOS ONE policies on sharing data and materials by including the following statement: "This does not alter our adherence to PLOS ONE policies on sharing data and materials.” (as detailed online in our guide for authors http://journals.plos.org/plosone/s/competing-interests). If there are restrictions on sharing of data and/or materials, please state these. Please note that we cannot proceed with consideration of your article until this information has been declared.  Please include your amended Competing Interests Statement within your cover letter. We will change the online submission form on your behalf. | Thanks for the suggestions. We included the following statement in the cover letter:  “Competing interests of Joachim Havla and Begüm Irmal Ön are:  JH: reports a grant for OCT research from the Friedrich-Baur-Stiftung, Amgen/Horizon, Sanofi, Roche, and Merck, personal fees and nonfinancial support from Merck, Alexion, Novartis, Roche, Celgene, Biogen, Bayer, Neuraxpharm and Horizon/Amgen, nonfinancial support from the Sumaira-Foundation and Guthy-Jackson Charitable Foundation, all outside the submitted work. This does not alter our adherence to PLOS ONE policies on sharing data and materials  BIÖ: Since February 2025, BIÖ is employed in Staburo GmbH, a data science company with clients in the biopharma industry. The projects and clients that BIÖ is involved in are all outside of the scope of the submitted work. This does not alter our adherence to PLOS ONE policies on sharing data and materials” |
| 4. Thank you for stating the following in the Competing Interests section:  [JH : reports a grant for OCT research from the Friedrich-Baur-Stiftung, Amgen/Horizon, Sanofi, Roche, and Merck, personal fees and nonfinancial support from Merck, Alexion, Novartis, Roche, Celgene, Biogen, Bayer, Neuraxpharm and Horizon/Amgen, nonfinancial support from the Sumaira-Foundation and Guthy-Jackson Charitable Foundation, all outside the submitted work.  BIO: Since February 2025, BIO is employed in Staburo GmbH, a data science company with clients in the biopharma industry. The projects and clients that BIO is involved in are all outside of the scope of the submitted work.].  We note that one or more of the authors are employed by a commercial company: Staburo GmbH.  1. Please provide an amended Funding Statement declaring this commercial affiliation, as well as a statement regarding the Role of Funders in your study. If the funding organization did not play a role in the study design, data collection and analysis, decision to publish, or preparation of the manuscript and only provided financial support in the form of authors' salaries and/or research materials, please review your statements relating to the author contributions, and ensure you have specifically and accurately indicated the role(s) that these authors had in your study. You can update author roles in the Author Contributions section of the online submission form.  Please also include the following statement within your amended Funding Statement.  “The funder provided support in the form of salaries for authors [insert relevant initials], but did not have any additional role in the study design, data collection and analysis, decision to publish, or preparation of the manuscript. The specific roles of these authors are articulated in the ‘author contributions’ section.”  If your commercial affiliation did play a role in your study, please state and explain this role within your updated Funding Statement.  2. Please also provide an updated Competing Interests Statement declaring this commercial affiliation along with any other relevant declarations relating to employment, consultancy, patents, products in development, or marketed products, etc.  Within your Competing Interests Statement, please confirm that this commercial affiliation does not alter your adherence to all PLOS ONE policies on sharing data and materials by including the following statement: "This does not alter our adherence to PLOS ONE policies on sharing data and materials.” (as detailed online in our guide for authors http://journals.plos.org/plosone/s/competing-interests). If this adherence statement is not accurate and there are restrictions on sharing of data and/or materials, please state these. Please note that we cannot proceed with consideration of your article until this information has been declared.  Please include both an updated Funding Statement and Competing Interests Statement in your cover letter. We will change the online submission form on your behalf. | Thanks for this suggestion.  We added the following to the Authors’ contributions section: “The commercial company Staburo GmbH, where BIÖ is employed since February 2025, was not involved in planning study design, data collection and analysis, decision to publish, or preparation of the manuscript and did not provide financial support in the form of authors' salaries and/or research materials.”  We added the following funding statement to the cover letter: “The funder Staburo GmbH provided support in the form of salaries for authors [BIÖ] but did not have any additional role in the study design, data collection and analysis, decision to publish, or preparation of the manuscript. The specific roles of these authors are articulated in the ‘author contributions’ section.”  We changed the competing interest Statment and incorporated it in the cover letter: “BIÖ: Since February 2025, BIÖ is employed in Staburo GmbH, a data science company with clients in the biopharma industry. The projects and clients that BIÖ is involved in are all outside of the scope of the submitted work. This does not alter our adherence to PLOS ONE policies on sharing data and materials” |
| 5. We noted in your submission details that a portion of your manuscript may have been presented or published elsewhere. [It exists an pre-print of an older version of the manuscript:  Buchka S, Joachim H, Begüm IÖ, et al. Individual level surrogacy of MRI T2 lesion information for future disease severity: a methodological discussion and application to recent MS Phase II and III trials.  The results presented in this manuscript will additionally published as a dissertation (monograph) of Stefan Buchka at the library of the Ludwig-Maximilians-University of Munich. The dissertation will not be publicly available before July 2026.]  Please clarify whether this conference proceeding or publication was peer-reviewed and formally published. If this work was previously peer-reviewed and published, in the cover letter please provide the reason that this work does not constitute dual publication and should be included in the current manuscript. | Thanks for this suggestion. We added the following statement to the cover letter: “Note, this article is based on the dissertation of Stefan Buchka. The dissertation (a monograph) was evaluated by an examination committee to confirm that Stefan Buchka could be awarded the doctoral degree. As part of this process, two independent external reviewers, who were members of the committee, also assessed the dissertation. To obtain the doctoral degree, the dissertation must be published in the library of the Ludwig-Maximilians-Universität in Munich. However, it will not become publicly available until July 2026, ensuring that there is no duplicate publication of the results. This embargo period can also be extended for an additional two years. Additionally, a preprint exists that has not been peer-reviewed.” |
| 6. In the online submission form, you indicated that [Data are only available by an application to CSDR (Clinical study data request: https://www.clinicalstudydatarequest.com/)].  All PLOS journals now require all data underlying the findings described in their manuscript to be freely available to other researchers, either 1. In a public repository, 2. Within the manuscript itself, or 3. Uploaded as supplementary information.  This policy applies to all data except where public deposition would breach compliance with the protocol approved by your research ethics board. If your data cannot be made publicly available for ethical or legal reasons (e.g., public availability would compromise patient privacy), please explain your reasons on resubmission and your exemption request will be escalated for approval. | We thank the editors for raising this important point. The data used in this study consists of individual-level data from complex phase II and phase III clinical trials. Public depositions of these data would breach compliance with the protocols approved by the respective research ethics boards, as longitudinal data combined with knowledge of the specific studies make full anonymization practically infeasible. Consequently, these data can only be accessed and analyzed within a controlled and secure research environment, such as through Clinical Study Data Request (CSDR). Unfortunately, we did not obtain explicit permission to publicly release the derived SEP and CEP datasets, but we comply fully with all ethical and legal requirements governing the handling of individual-level data. We added the following statement to the Availability of data and materials section (lines 622 - 626): “The data underlying this study originate from phase II and III clinical trials and cannot be publicly shared due to ethical and privacy restrictions. The longitudinal and study-specific nature of the data prevents full anonymization. Data access is therefore restricted to controlled and secure research environments and can be requested via Clinical Study Data Request (CSDR).” |
| 7. Your ethics statement should only appear in the Methods section of your manuscript. If your ethics statement is written in any section besides the Methods, please move it to the Methods section and delete it from any other section. Please ensure that your ethics statement is included in your manuscript, as the ethics statement entered into the online submission form will not be published alongside your manuscript. | Thanks for this suggestion. We moved the ethic section to the material and methods part. |
| 8. Please include captions for your Supporting Information files at the end of your manuscript, and update any in-text citations to match accordingly. Please see our Supporting Information guidelines for more information: [http://journals.plos.org/plosone/s/supporting-information](http://track.editorialmanager.com/CL0/http:%2F%2Fjournals.plos.org%2Fplosone%2Fs%2Fsupporting-information/1/010f0199559d055a-51225b04-92db-46b0-a283-de095b17c436-000000/o598ni7nLYOI6KVL0iBCjvKOAwuh4HNsomE6Rlw77kw=228). | Thanks for this suggestion. We re-placed this section. |
